# Biological and Teratogenic Evaluations of Nitrogen Heterocycles for Anticancer Therapy

**DOI:** 10.3390/ph19030405

**Published:** 2026-03-01

**Authors:** Jéssica Celerino dos Santos, Josival Emanuel Ferreira Alves, Rafael David Souto de Azevedo, Josefa Gerlane da Silva, Maria Regina de Oliveira Silva, Lucia Patrícia Bezerra Gomes da Silva, Caio Victor Silva Soares, Jamire Muriel da Silva, Nabuêr Francieli da Silva, Jamerson Ferreira de Oliveira, Maria do Carmo Alves de Lima, Ricardo Olímpio de Moura, Sinara Mônica Vitalino de Almeida

**Affiliations:** 1 Molecular Biology Laboratory, University of Pernambuco (UPE), Multicampi Garanhuns, Garanhuns 55294-902, PE, Brazilsinara.monica@upe.br (S.M.V.d.A.); 2 Keizo Asami Immunopathology Laboratory (LIKA), Federal University of Pernambuco, Recife 50670-901, PE, Brazil; 3 Institute of Health Sciences, University of International Integration of Afro-Brazilian Lusophony (UNILAB), Redenção 62790-970, CE, Brazil; 4 Chemistry and Therapeutic Innovation Laboratory (LQIT), Department of Antibiotics, Federal University of Pernambuco, Recife 50670-901, PE, Brazilmaria.calima@ufpe.br (M.d.C.A.d.L.); 5 Department of Pharmacy, Laboratory of Synthesis and Vectorization of Molecules, State University of Paraíba (UEPB), Campina Grande 58429-500, PB, Brazil; ricardo.olimpiodemoura@servidor.uepb.edu.br

**Keywords:** acridine, quinoline, DNA binder, *Danio rerio*

## Abstract

**Background**: Heterocycle compounds with acridine, quinoline, indole, and pyridine nuclei are potentially active for anticancer activity since they can promote inhibition of vital enzymes, decreasing cell survival after binding to biomolecules. However, unspecific biological interactions can result in unwanted effects, which should be defined during the synthesis and proposition of new molecules. Thus, the objective of this study was to investigate the biological and teratogenic effects of four nitrogen heterocycles proposed for anticancer therapy. **Methods**: Four 2-cyano-*N*-phenylacrylamine type derivatives containing acridine (**3a**), quinoline (**3b**), indole (**3c**), and pyridine (**3d**) nuclei were synthesized and characterized. They were evaluated for their ability to interact with DNA, physicochemical and pharmacokinetic predictions, in vitro and in silico methodologies, besides in vitro inhibition of the Topoisomerase IIα enzyme, antiproliferative activity in tumor and non-tumor cells, hemolytic activity with human erythrocytes, and in vivo toxicological studies with zebrafish embryos. **Results**: UV–vis absorption studies with ssDNA revealed different spectroscopic effects, with binding constants (Kb) ranging from 1.41 × 10^5^ to 6.46 × 10^4^ M^−1^. The fluorescence quenching constant (Ksv) with ethidium bromide (EB) varied between 0.53 and 0.67 × 10^3^ M^−1^. The compounds intercalated into DNA base pairs, a mechanism confirmed by molecular docking, with **3b** (quinoline) showing the most substantial interaction. All derivatives exhibited antitopoisomerase IIα activity at 100 μM and were cytotoxic against MCF-7 and T47-D breast tumor cells, particularly against the more aggressive T47-D lineage. No hemolytic activity was observed in human erythrocytes. In vivo assays in zebrafish embryos showed no toxicological or cardiotoxic effects. However, all compounds altered superoxide dismutase (SOD) and catalase (CAT) enzymatic activity, requiring further studies on reactive oxygen species (ROS) generation to assess potential adverse effects. Furthermore, significant results were observed in the physicochemical and pharmacokinetic parameters of the synthesized compounds. **Conclusions**: The findings highlight the quinoline derivative (**3b)** as the most promising nitrogen heterocycle due to its antiproliferative activity and biomolecular interactions without adverse effects in zebrafish embryos, distinguishing it from clinically available agents.

## 1. Introduction

Cancer continues to be a persistent public health challenge with increasing numbers on a global scale [1]. The resistance of tumor cells to available drugs and adverse effects drives the search for new antitumor medications, aiming to slow tumor growth, eliminate cancerous cells, and reduce unwanted side effects. In this context, organic compounds that contain nitrogen heterocyclic skeletons (N-heterocycles) are promising due to their favorable pharmacological properties [2,3,4,5,6].

Among the options for new drug candidates, nitrogen heterocycles based on acridine, quinoline, indole, and pyridine nuclei stand out because they exhibit cytotoxic activity against tumor cells, the ability to interact with DNA, and the inhibition of the topoisomerase enzyme [7]. These mechanisms of action are described by the works of Almeida et al. [8] with spiro-acridine derivatives, Ribeiro et al. [9] with quinoline derivatives, Alves et al. [10] with indole derivatives, and Narva et al. [11] with pyridine derivatives. These different nuclei have proven promising for cancer therapy by targeting key molecules and inducing the death of tumor cells.

Almeida et al. [8] produced new spiro-acridines by introducing a cyano-*N*-acyl hydrazone group between the acridine and (non-methoxy-substituted) phenyl rings, followed by spontaneous cyclization. The compounds were specific for human topoisomerase IIα inhibition and retained the DNA interaction ability. Recently, Santos et al. [7] synthesized four new nitrogen heterocyclic derivatives containing the acridine, quinoline, indole, and pyridine nuclei and studied them by in vitro and in vivo tests, finding that the indole ring favored biological activity. Indole derivatives exhibited the highest DNA binding constant (Kb) of 9.54 × 10^4^ and fluorescence quenching (Ksv) of 0.64 × 10^3^ M^−1^, the best topoisomerase enzyme inhibition profile with 82% inhibition of enzymatic activity, and cytotoxic activity against tumor cells, without side effects for the zebrafish model [7].

For new anticancer molecule propositions, candidate drugs can be explored through interaction studies involving biomolecules such as DNA (the primary target for cancer therapy) [12]. Additionally, zebrafish (*Danio rerio*) have proven to be a valuable tool in anticancer research, providing crucial information on the efficacy and toxicity of new compounds to assess possible adverse effects and determine the best therapeutic dose of these compounds [13,14].

**Scheme 1:**
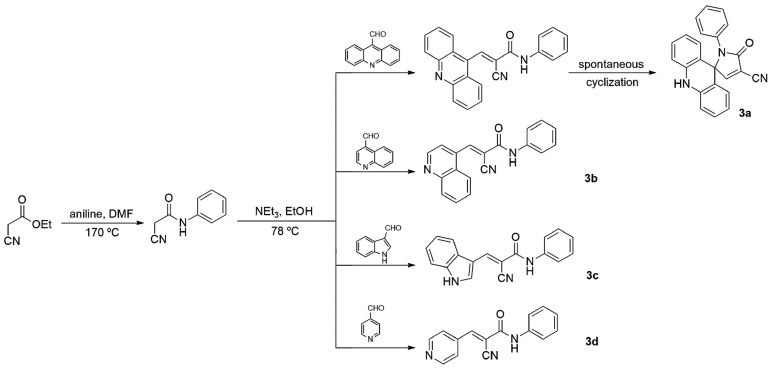
Synthesis of 2-cyano-*N*-phenylacrylamide derivatives.

Based on the previous results for the indole derivative described by Santos et al. [7] and the data of Almeida et al. [8] for the spiro acridines, beyond the therapeutic potential of the quinoline ring [9,15] well reported in the literature, four nitrogen heterocyclic derivatives with different nuclei were tested here (Scheme 1): [5-oxo-1-phenyl-1,5-dihydro-10*H*-spiro(acridin-9,2-pyrro)-4-cya nitrile (**3a**—acridine)], 3-(quinoline-4yl)-2-cyano-*N*-phenylacrylamide (**3b**—quinoline), 2-cyano-3-(1*H*-indol-3yl)-*N*-phenyl acrylamide (**3c**—indole), and [3-(pyridine-4yl)-2-cyano-*N*-phenylacrylamide] (**3d**—pyridine). The molecules were designed to improve biological activity and to reduce adverse effects on non-tumoral cells, as well as on in vivo organisms, in an effort to contribute to the advancement of studies focused on cancer therapy. The compounds were characterized and evaluated for their ability to interact with DNA, for physicochemical and pharmacokinetic predictions using in vitro and in silico methodologies, as well as for in vitro inhibition of the Topoisomerase IIα enzyme, antiproliferative activity in tumor and non-tumor cells, hemolytic activity with human erythrocytes, and in vivo toxicological studies with zebrafish embryos. Thus, the objective of this study was to investigate the biological and teratogenic effects of nitrogen heterocycles for anticancer therapy.

## 2. Results and Discussion

### 2.1. Nitrogen Heterocyclic Compounds

Studies on spiroacridine derivatives obtained through spontaneous cyclization of the parent acridine compound were reported by the Almeida group. Reference [8] raised interest in synthesizing new nitrogen heterocyclic derivatives with spirocyclization using the same side chemical portion, but with the cyano-acetamide spacer instead of the cyano-acylhydrazone. The present spacer spontaneously cyclized only in the acridine derivative (**3a**), as demonstrated in Scheme 1. The three other compounds were synthesized: quinoline (**3b**), indole (**3c**), and pyridine (**3d**). The synthesis of **3b** was previously described by Silva et al. [16].

As established by previous studies, it was observed that 9-acridinecarbaldehyde, 4-pyridinecarbaldehyde, 4-quinolinecarbaldehyde, and 3-indolecarbaldehyde reacted effectively in absolute ethanol in the presence of triethylamine with their respective intermediates, 2-cyano-*N*’-phenylacetamide, through a Knoevenagel condensation reaction [8,17]. The condensation reaction resulted in the formation of the corresponding compounds **3a**–**3d**. The 9-acridinecarbaldehyde underwent spontaneous cyclization between the N1 group of the acetamide and the C-9 carbon of the acridine. Regarding the yields of the final compounds, they were found to be above 50% (**3a**–**3c**) and 32% (**3d**).

The structures of the four derivatives (**3a**–**3d**) were confirmed by ^1^H NMR, ^13^C NMR, mass spectrometry, infrared spectroscopy, and high-resolution spectroscopy. The ^1^H NMR analysis allowed for the identification of distinct features, such as the singlets of the amide protons (NH) with chemical shifts ranging from δ 10.54 to 9.63 ppm. Additionally, the ^1^H NMR spectra of all derivatives revealed singlet (s), doublet (d), triplet (t), and multiplet (m) signals associated with the acridine aromatic groups, confirming the presence of a ring similar to that described in the literature [8,18]. Furthermore, the ^13^C NMR spectra corroborated the structures, as they presented signals corresponding to the carbon atoms. Exclusively in acridine compound (**3a**), a shift at 70.6 ppm was observed, indicating the formation of the quaternary carbon after the process of spontaneous cycling. Particularly, the derivative **3a** showed a singlet at 9.63 ppm corresponding to the NH of the acridine nucleus, which is indicative of the cyclization as previously described by Albino and coworkers [18]. This mechanism was first reported by Vilková et al. [19]. Cyclization occurs spontaneously, without the need for additional energy. Our theoretical results confirm that cyclization is energetically favored, as the cyclic isomer possesses lower energy (10 kJ mol^−1^) compared to the open isomer, since molecules tend to adopt a lower energy form, rendering them more stable.

The results of the infrared spectra also supported the structural characterization of the new compounds (**3a**–**3d**). The following bands were observed: 3314–3470 cm^−1^, suggestive of axial deformation of NH; a band between 3019 and 3262 cm^−1^, suggestive of axial deformation of CH; and a band between 1640 and 1695 cm^−1^, suggestive of aromatic axial deformation of C=O [8,20]. Additionally, mass spectrometry (MS) was useful in the structural characterization of the new nitrogenous derivatives. These data are presented in the Supplementary Material.

### 2.2. Physicochemical and Pharmacokinetic Characteristics of the Compounds

Prior to the in vitro and in vivo evaluations, druglikeness studies generated by SwissADME and multi-parameter optimization (MPO) obtained using Marvin allowed visualization of the favorable physicochemical profile of the compounds (Table 1). Based on the results generated by SwissADME, all compounds exhibit favorable characteristics according to Lipinski’s rule of five [21], Ghose’s rule [22], Veber’s rule [23], Egan’s rule [24], and Muegge’s rule [25], indicating their potential as drug candidates. Furthermore, regarding the multi-parameter optimization (MPO) system, which integrates drug candidates and active drugs, as indicated by Wager et al. [26], all compounds presented stable pharmacokinetic profiles, with MPO values above 4.0, indicating favorable passive permeability (Papp), low efflux by P-glycoprotein (P-gp), and low metabolic clearance.

**Figure 1:**
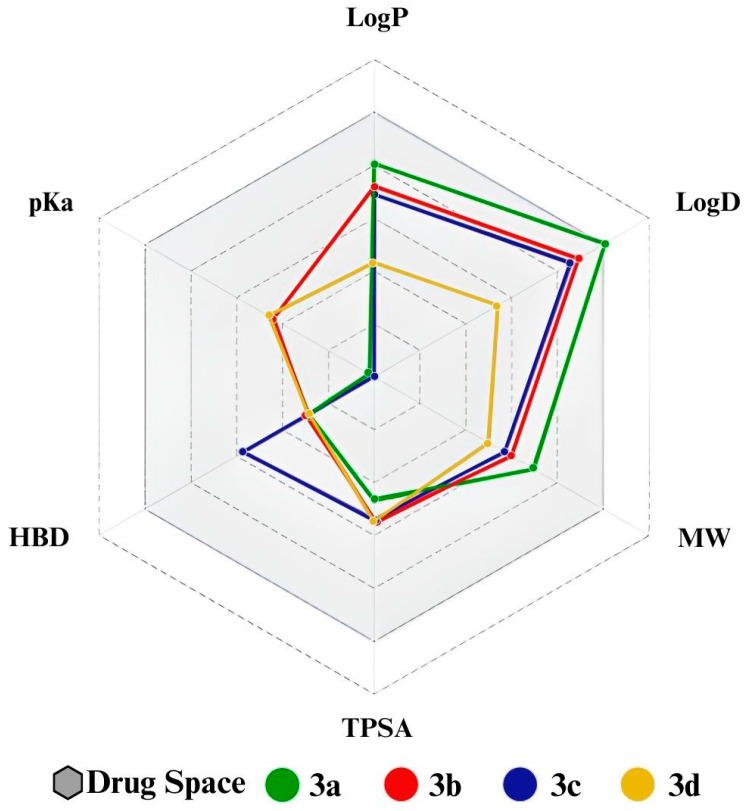
Pharmacokinetic feasibility radar using the MPO algorithm in relation to Pfizer’s drug space (space in gray).

**Figure 2:**
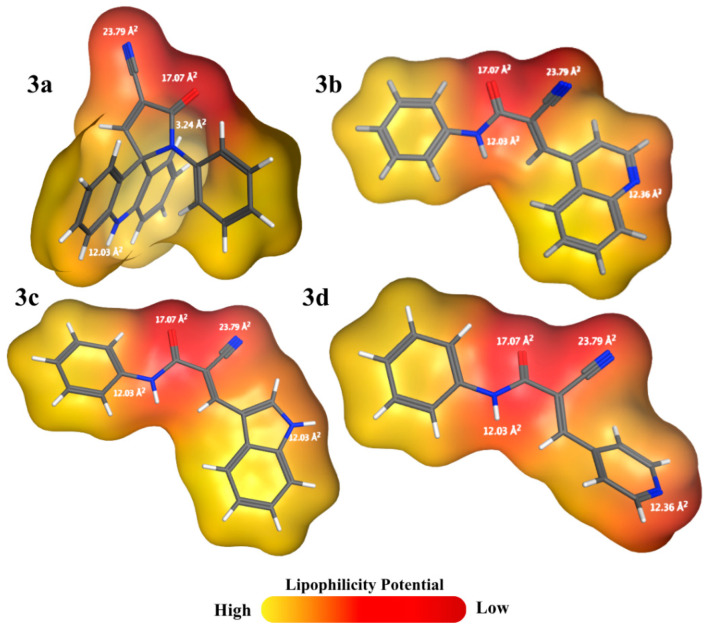
Map of molecular lipophilicity potential (MLP) for compounds **3a**, **3b**, **3c**, and **3d**.

Although all compounds met druglikeness requirements and presented properties within the ideal range (Figure 1), compound **3d** obtained the highest MPO value (4.67). This condition occurs because the LogP and LogD values (2.16) are close to the values considered ideal (LogP ≤ 3 and LogD ≤ 2), since **3d** consists of the smallest structure (249.27 g/mol) and is composed of important polar groups. However, all molecules have favorable and similar TPSA results (Figure 2), mainly associated with carbonyl, nitrile, and *N*-substituted amide groups, which are present in all compounds [27]. Furthermore, **3c** and **3d** also have an imine nitrogen contributing to the polar area and have the same TPSA value (65.45 Å^2^), which is close to the result for **3b** (64.98 Å^2^). However, because **3b** and **3c** have an indole and quinoline nucleus, respectively, they have a larger size (299.33 g/mol and 287.32 g/mol, respectively) and lipophilicity (3.54 and 3.48, respectively) than **3d**, which has a pyridine group as a substituent.

Furthermore, compound **3a** obtained the lowest MPO value (4.24), as it is the compound with the highest lipophilic character, presenting the highest LogD and LogP results (4.03 for both), which is associated with the presence of the acridine group, which also leads to an increase in molar mass (348.49 g/mol) and molar refractivity (MR) (110.26). In addition, it is evident that it is the compound with the lowest TPSA (Figure 2), due to the *N*-substituted amide in the structure binding to acridine to form a tertiary amine, which has a lower contribution to the polar area. It can also be seen that the formation of this bond also promotes a conformational restriction, causing **3a** to have only one rotatable bond (RB), while the others have four [26,27].

Regarding the pKa values (Table 1), it is possible to classify all of them as low-potential bases, as they have strong conjugate acids, with pKa values below 5.0. This is because, despite having *N*-substituted amides and a tertiary amine, which act as bases, in all molecules, there is a displacement of the electronic density of these groups due to the mesomeric and/or steric effect, causing the base to have its electrons displaced throughout the structure. However, **3a** and **3c** have much lower pKa values because the free electrons of nitrogen in acridine and indole are actively participating in resonance, which promotes greater stabilization of the base. Meanwhile, in derivatives **3b** and **3d**, the free electrons are more available for protonation, making them weaker acids than **3a** and **3c**.

Based on the determination of pharmacokinetic parameters using ADMET Lab 3.0, the results presented in Table 2 can be interpreted as follows. When highlighting the permeability process, all compounds have greater permeability compared to etoposide, which has a large number of polar groups and a high molecular mass of 588.55 g/mol. Furthermore, **3a**, **3b**, and **3c** have high permeability via Caco-2 (6.60 × 10^−5^ cm/s, 1.11 × 10^−5^ cm/s, and 1.15 × 10^−5^ cm/s, respectively) and MDCK (2.23 × 10^−5^ cm/s, 1.48 × 10^−5^ cm/s, and 1.45 × 10^−5^ cm/s), while **3d** has high permeability only for MDCK (1.72 × 10^−5^ cm/s), being moderate for Caco-2 (8.57 × 10^−6^ cm/s) [28]. It should be noted that this prediction is associated with the superior lipophilic potential of compounds **3a**, **3b**, and **3c** in relation to **3d**, which is also considered the smallest structure in relation to the others, having the lowest molar mass.

Furthermore, by understanding the distribution processes, it can be highlighted that derivative **3a** has the greatest ability to penetrate tissues (1.08 L/kg), presenting the largest volume of distribution, in addition to having one of the most significant probabilities of crossing the blood–brain barrier (5.7%). However, in general, all have a strong tendency to remain in plasma, as they present distribution volume results lower than 2 L/kg, with **3b** (0.59 L/kg), **3c** (0.50 L/kg), and **3d** (0.56 L/kg) presenting very low values in relation to **3a**. This condition may be associated with a strong tendency for molecules to bind to plasma proteins, as they all have results above 90%. Regarding BBB permeability, with the exception of **3a**, all have results below 1%, which may be associated with the greater hydrophilic character of the compounds [29,30].

Regarding excretion, there is an increase in plasma clearance from **3a** to **3d**, due to an increase in the polarity of the molecule and a decrease in lipophilicity and molar mass. Thus, **3c** (0.30972 L/h/K) and **3d** (0.30222 L/h/K) have moderate clearance, while **3a** (0.2208 L/h/kg) and **3b** (0.2772 L/h/K) have low clearance. However, among the four compounds, only **3a** has a relatively high half-life (3h 23min), while the others vary between 1 and 2 h. This is because, in addition to having low clearance, compound **3a** has the largest volume of distribution, which makes it the least available for metabolism and excretion [31].

According to the first-phase metabolism process, based on the results generated by *ADMETlab 3.0* and *Xenosite* evaluated in Table 3, CYP1A2, CYP2C19, and CYP3A4 can be identified as important enzymes involved in the metabolism of the compounds. CYP1A2, a priori, proves to be effective in catalyzing reactions in **3a** (82.1%), **3c** (63.2%), and **3d** (97.9%), promoting hydroxylation primarily in aromatic structures of neutral lipophilic planar substrates. Similarly, CYP2C19, which is presented as being capable of metabolizing only **3a** (100%), also performs hydroxylation in aromatic and aliphatic structures of lipophilic, neutral, and intermediate-sized substrates. Finally, CYP3A4 promotes the metabolism of **3a** (100%) and **3d** (59.5%) in greater proportions, performing the oxidation of aliphatic structures, with a greater preference for large, lipophilic molecules [32,33].

**Figure 3:**
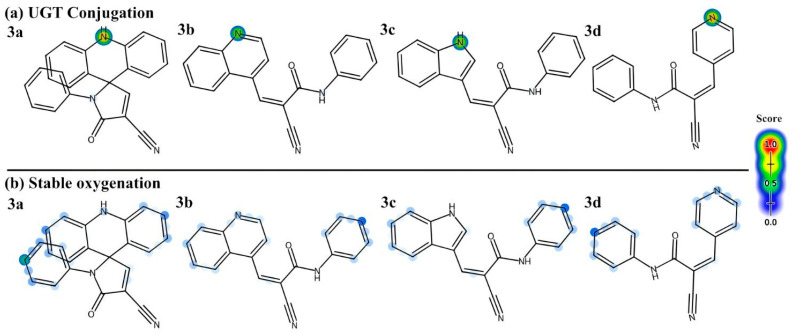
(**a**) Metabolization points by glucuronidation. (**b**) Metabolization points by stable oxidation for the compounds **3a**, **3b**, **3c**, and **3d**.

This explains the strong tendency for oxidation reactions to form with the compounds, as shown in Figure 3. However, it is understood that compound **3b** has a low probability of being metabolized by any of the enzymes mentioned, despite its structural similarity to the other compounds. Furthermore, it can also be seen that the amide group in compounds **3b**, **3c**, and **3d** may be targeted by hepatic peptidases, resulting in a phase I hydrolysis reaction that leads to the formation of carboxylic acids and amines. In addition, among phase II reactions, predictions generated by Xenosite indicate a strong tendency toward the formation of conjugates, resulting in glucuronic complexes. In this context, the nitrogen atoms present in the cyclic structures act as important nucleophiles in the reaction. However, only compounds **3a** and **3c** present secondary amines, which exhibit greater reactivity toward conjugation. In contrast, **3b** and **3d,** tertiary amines stand out, which are less reactive and require prior oxidation steps to undergo glucuronidation [34].

**Figure 4:**
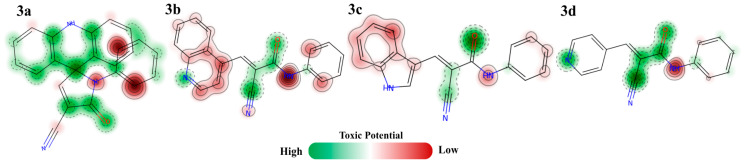
Toxicity points generated by STopTox for the compounds **3a**, **3b**, **3c**, and **3d**.

Finally, among the toxicity criteria evaluated by ProTox 3.0, it is possible to predict that only compound **3a** shows cardiotoxic potential, with a probability of 92%, while for other predictions, such as nephrotoxicity, carcinogenicity, mutagenicity, and cytotoxicity, it is inactive. For the other compounds, a favorable safety profile is understood. Only a slight potential for hepatotoxicity is presented, but no compound has a probability of greater than 60%, as shown in Table 4. Comprehensively, with the detection of toxicity points (Figure 4), based on the predictions generated by STopTox, it is possible to identify amine groups and aromatic portions associated with the toxic potential of the compounds. While carbonyl and nitrile nuclei are generally less toxic regions, for **3a**, the less toxic potential of acridine substitution is understood. It can be observed that compound **3a** has the most intense toxicity points, associated with the *N*-phenyl portion, which may be related to its higher probability of cardiotoxicity. In relation to hepatotoxicity, among the main associated aspects, it is possible to predict the ability of molecules to be metabolized by enzymes that make up the CYP450 system, which has been observed to occur, as well as the daily dose administered. Another important factor commonly associated with hepatotoxicity is lipophilicity, but the relationship between the LogP of the compounds and the results obtained is not understood [35].

### 2.3. DNA Interactions

Biological activities and toxic effects of nitrogen heterocyclic derivatives can be caused by the ability to bind [36]. Therefore, the compounds **3a**, **3b**, **3c**, and **3d** were subjected to absorption analysis in the absence and presence of ssDNA. Spectroscopic analyses revealed absorption bands ranging from 271 to 420 nm. The maximum absorption wavelengths of the compounds in their free and bound forms, along with other spectrophotometric data of the derivatives, are available in Table 5. In the presence of increasing amounts of ssDNA (0 to 100 μM), changes in the absorption spectra were observed, exhibiting hypochromic and hyperchromic effects, with shifts in the maximum absorption peaks towards the blue (hypochromic) and red (bathochromic) regions (Table 5 and Supplementary Material Figure S17). When it comes to the interaction of drug candidates with biomolecules, spectral changes and shifts in the maximum absorption peaks indicate the formation of ligand–DNA complexes [37,38,39,40].

The compounds **3a**, **3b**, and **3d** demonstrated hypochromic effects in the presence of ssDNA, with varying degrees of absorption reduction. The highest hypochromic effect was 91.63%, observed for the quinoline derivative (**3b**). The presence of the derivatives contributed to the spectral changes, stabilizing the DNA structure and resulting in hypochromism, indicating an intercalative type of interaction [41]. In general, the combination of hypochromism and bathochromism is due to the insertion of the molecules between the DNA base pairs, establishing hydrogen bonds [41,42]. The characteristics were particularly notable for the compounds **3a** (spiroacridine) and **3b** (quinoline), demonstrating a shift in the absorption peak towards the red region (bathochromic effect), with values of 71 nm and 78 nm, respectively. Regarding the binding affinity constant (Kb), values ranged from 5.26 × 10^4^ to 6.46 × 10^5^ M^−1^ (Table 5). The highest Kb value was observed for **3a** (acridine). The Kb values found for the derivatives are characteristic of intercalators, which are often in the range between 10^4^ and 10^6^ M^−1^ [36].

In the development of new antitumor drugs, understanding and elucidating the mechanism of interaction of molecules with DNA is essential [43]. Absorption studies provide valuable information about the binding mechanism of compounds to DNA. However, for a better understanding, competition studies with DNA intercalating probes, such as ethidium bromide (EB), are necessary, as they already have their binding mode well established [41]. In this competition assay, we used EB as the DNA intercalating probe: EB-ssDNA, given that the fluorescence intensity of isolated DNA is weak. The assay was conducted both in the absence and presence of the derivatives **3a**, **3b**, **3c,** and **3d,** with the emission detailed in Table 6.

EB is a fluorophore when intercalated with DNA, serving as a probe for the DNA complex formation [44]. As presented in Table 6, the fluorescence intensity of the EB-ssDNA complex exhibited its maximum emission peak at 599 nm. After the addition of the compounds, the fluorescence signal of the EB-ssDNA complexes decreased as the concentrations of the compounds increased, in which the fluorescence suppression of the probe occurs due to the probe’s fluorophore being replaced by another intercalating agent, resulting in the displacement of the probe and suggesting a more intense interaction between the compounds and DNA [42,44,45].

**Figure 5:**
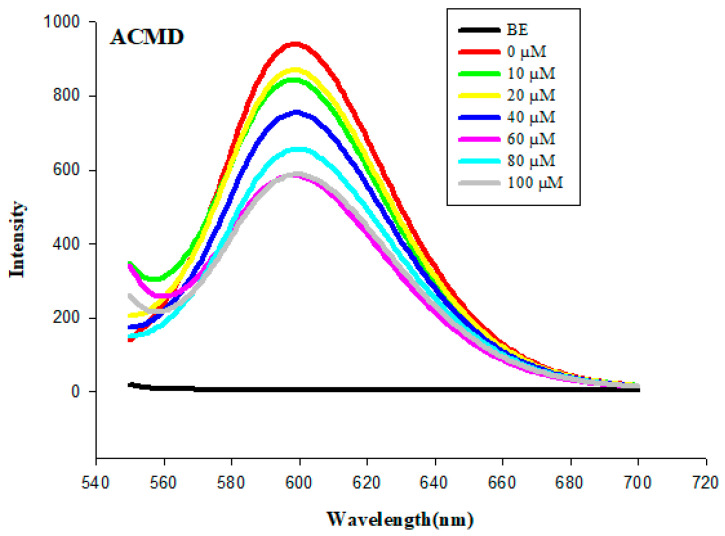
Emission spectra of free and ssDNA-bound EB, in the absence and presence of **3a** derivative.

The fluorescence quenching values of the EB-ssDNA complex were calculated, with the lowest fluorescence quenching percentage being 53.29% for **3c** (indole) and the highest percentage of 73.14% for **3b** (quinoline). This indicates that the compounds compete with the probe and intercalate the DNA base pairs. Regarding the fluorescence quenching profile of the compounds, the Stern–Volmer equation was used to calculate the fluorescence quenching constant (Ksv) (Table 6). The highest Ksv values for EB-ssDNA were observed for **3a** (acridine) with 6.7 × 10^2^ M^−1^. These data confirm the findings in the UV–vis absorption assay, as these same derivatives were the ones that presented the highest binding affinity constants (Kb), with acridine being particularly notable (Figure 5).

Competition studies using DNA intercalating probes such as EB were conducted by Santos et al. [7]. Their findings indicated that derivatives with different nuclei (acridine, quinoline, indole, and pyridine) caused fluorescence quenching of EB. Similarly to our findings, the fluorescence quenching values ranged from 0.34 to 0.64 × 10^3^ M^−1^ for EB. In other words, the results of the present study consolidate the data previously obtained by the research group, although they present different side groups. Furthermore, the data regarding intercalation between DNA base pairs corroborate the findings [20,45,46,47], which studied compounds with acridine, spiroacridine, indole, and pyridine nuclei, respectively, and attributed the ability to intercalate DNA base pairs to the planarity of polyaromatic compounds.

### 2.4. Human Topoisomerase Inhibition Assay

Considering that topoisomerase enzymes are involved in vital processes for DNA regulation, such as proliferation, differentiation, cell survival, and maintenance of genome integrity, various studies have conducted antitumor therapeutic investigations due to their functions [20,48]. Topoisomerase IIα (topo) is one of the main therapeutic targets due to its overexpression during the proliferation process of tumor cells [49]. In its mechanism of action, topoisomerase inhibitors cause damage to tumor cells by promoting DNA cleavages while simultaneously inhibiting the formation of new DNA strands, thereby activating the cell death process (apoptosis) [50,51,52].

**Figure 6:**
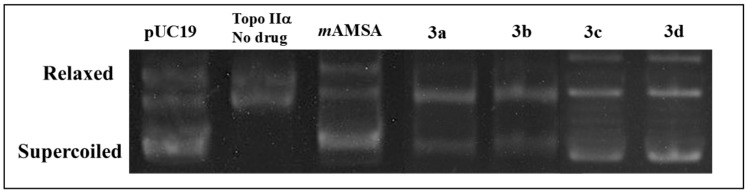
Effect of m-AMSA, **3a**, **3b**, **3c**, and **3d** at 100 μM in the Topo IIα enzymatic inhibition assay.

The experimental assay was conducted to evaluate the effects of the derivatives against human topo IIα. In Figure 6, alterations in the migration of DNA bands (pUC19) are observed, representing different structural arrangements such as supercoiled DNA, circular and relaxed DNA, and linear DNA [46,53]. The inhibition of Topo IIα was analyzed based on the bands corresponding to supercoiled DNA (pUC19) in relation to the positive control. The derivatives **3a** (acridine), **3b** (quinoline), **3c** (indole), and **3d** (pyridine) inhibited the activity of Topo IIα at 100 μM. Quantitatively, the positive control m-AMSA showed 100% inhibition, while the derivatives **3a**, **3b**, **3c**, and **3d** exhibited inhibitions of 77%, 82%, 93%, and 98%, respectively.

The appearance of linear DNA bands indicates that the compounds acted as Topo IIα poisons [20]. In addition to the formation of linear DNA, the topoisomerase were observed, suggesting the formation of a cleavage complex between Topo IIα, pUC19, and the tested compounds. The presence of this complex indicates that the compounds were effective in inhibiting the enzyme and can maintain DNA cleavage, thereby preventing cellular replication in tumor cells [46].

Previous to our study, Gouveia et al. [20] demonstrated with spiroacridine derivatives an inhibition percentage of 61.70% at 100 μM, a result similar to our findings for **3a**, which inhibited the enzyme by 77% at 100 μM. In accordance with our findings, Silva Filho et al. [53] conducted enzymatic inhibition tests of Topo IIα using two acridine-thiosemicarbazone derivatives and observed that those substituted with phenyl and naphthyl groups promoted inhibition of enzymatic activity at 100 μM, with the best result for the derivative bearing the phenyl substituent on the side chain. Furthermore, in line with the findings of Santos et al. [7], the derivatives with indole and pyridine rings exhibited 82% and 80% inhibition of the topoisomerase enzyme at the same concentration tested here. However, our inhibition values were even higher, at 93% and 98%, respectively, indicating that the substitution of the amide nitrogen with a phenyl group in the different nuclei has enhanced their biological activity.

**Figure 7:**
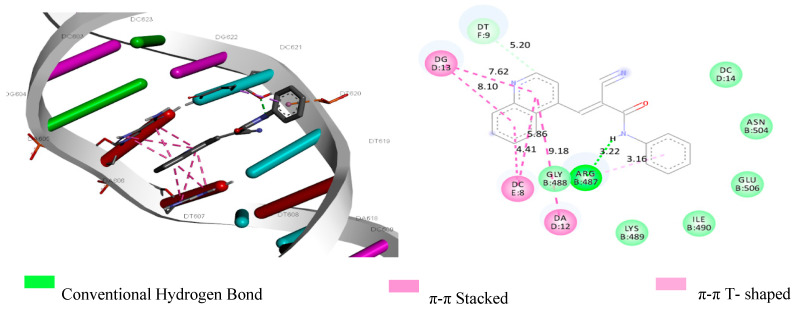
Docking poses of the **3b** derivative with their respective interaction modes with DNA and amino acid residues of the topoisomerase active site, including hydrogen bonding interactions and π-π stacking interactions of the derivative’s aromatic ring with nitrogenous bases A12-D, C8-E, and G13-D.

In addition to the spectroscopic studies of the in vitro interaction between the derivatives and DNA at the topoisomerase action site, computational studies of molecular docking and molecular dynamics were also conducted to corroborate the in vitro studies. Molecular docking studies have the capability to predict the binding conformation of molecules and assist in the discovery of new molecules capable of binding and acting on biological targets, such as DNA and topoisomerase IIα [9,54,55]. The binding energy data of the derivatives studied are presented in Table 7, and the docking poses are depicted in Figure 7.

As presented in Table 7, all derivatives exhibited negative binding energy values, with these values being lower than that of the ligand 9-acridine, which showed a binding energy of −6.99 kcal mol^−1^. The binding energy values of the derivatives with the target 1G3X, in ascending order, were **3d** (−7.35), **3a** (−8.07), **3c** (−8.75), and **3b** (−9.23) kcal mol^−1^. The same sequence was observed for the target 5GWK: **3d** < **3a** < **3c** < **3b**, with **3b** (quinoline) having the lowest binding energy value (−8.84 kcal mol^−1^) for target 5GWK and (−9.23 kcal mol^−1^) for target 1G3X. The binding pose of the ligand with the lowest binding energy, **3b**, indicates that there was an intercalative interaction between the DNA nitrogenous base pairs and that the ligands interact with the amino acid Arg-487 in a manner similar to etoposide (a co-crystallized drug present in the topo IIα structure), as observed in Figure 7. Additionally, **3a** and **3b** exhibited π-stacking interactions between the DNA base pairs and their aromatic rings; π-stacking interactions are essential in the intercalative interaction process [10].

The binding energy values confirm the findings from the in vitro assays, as **3b** exhibited the second-highest Kb and Ksv values in the competition tests with EB, and the compounds demonstrate a higher binding affinity within the ligand–receptor complex, indicating a stronger and more stable interaction.

### 2.5. Antiproliferative and Hemolytic Evaluations

In this study, using the sulforhodamine assay, the antiproliferative activity of the compounds **3a**, **3b**, **3c**, and **3d** was evaluated in tumor and non-tumor cells, with MCF-7 and T47-D being the tumor cell lines and HaCaT the non-tumor cell line. The compounds were tested at different concentrations, specifically 0.25, 2.5, 25, and 250 µM, with *m*-AMSA used as a positive control. The GI_50_ values (the molar concentration of the compound that inhibits 50% of cell growth) for both cell lines are presented in Table 8.

Cancer therapy faces a constant therapeutic challenge, as drugs often do not act specifically against tumor cells, being cytotoxic also to healthy cells, including human erythrocytes, because cytotoxicity can cause damage to the cell membrane and immediate cell death [37,40]. For this reason, a hemolytic assay was conducted to evaluate possible damage to the cell membranes of human erythrocytes. According to the results in Table 8, the four compounds with different nuclei presented hemolysis rates below 7%, meeting the toxicity criterion (<10%). In other words, none of the compounds caused significant hemolysis in human erythrocytes, even at the highest tested concentration of 100 µM [56].

As presented in Table 8, the GI_50_ values varied among the compounds and the cell lines. The GI_50_ values for the tumor cell lines ranged between 3.42 and 34.41 µM. The compounds **3a** and **3b** exhibited higher cytotoxicity for the T47-D tumor cell line with GI_50_ values of 3.42 and 8.07 µM, respectively. Acridine derivative was cytotoxic against MCF-7 (34.41 μM) and T47-D (3.42 μM) cell lines, showing greater inhibition of cell growth for T47-D. This same compound showed the highest GI_50_ in HaCaT (>100 μM), indicating low cytotoxicity in non-tumor cells and higher antiproliferative activity in tumor cells, especially T47-D. Among the tested compounds, the highest hemolytic activity of 6.66% was exhibited by the acridine derivative (**3a**) at the highest concentration (100 µM), still being considered non-hemolytic. In turn, the reference drug *m*-AMSA showed a hemolytic activity of 19% at the highest concentration. This should be due to the lateral chemical groups (methoxy) present on the reference acridine that result in a lower cell inhibition concentration but cause well-described side effects, which justify the ongoing search for new acridine derivatives, as reported here and in the literature [4,7,8,18].

Antiproliferative effects of phenyl-substituted acridine-thiosemicarbazone derivatives against the MCF-7 and HaCaT cell lines were described by Almeida et al. [36]. The authors reported that the most active compound inhibited the growth of all cell lines with GI_50_ values below 10 µM for all tumor cell lines, including the non-tumor HaCaT line. In contrast, our compound **3a** (acridine) with a substituted *N*-phenyl amide was not as active against HaCaT cells and was cytotoxic to MCF-7 with a GI_50_ of 34.41 μM.

In turn, the quinoline derivative **3b** exhibited GI_50_ values of 33.62 μM for the MCF-7 cell line and 2.98 μM for HaCaT, showing low selectivity between tumor cells and greater selectivity for non-tumor cells and the T47-D line. However, in the HaCaT line, our compounds showed higher GI_50_ values compared to the m-AMSA control. Perković et al. [39] and Santos et al. [7] investigated the antiproliferative activity of compounds containing pyridine, indole, and quinoline heterocyclic structures. Perković et al. [39] reported the results of the compounds against the MCF-7 tumor cell line and determined GI_50_ values (μM) ranging from 2.0 ± 0.1 to 11.0 ± 0.3, a result like that of **3c** (indole). Santos et al. [7] studied different nitrogen heterocyclic nuclei (acridine, quinoline, indole, and pyridine) and conducted studies with tumor cell lines, including MCF-7, T47-D, and the non-tumor HaCaT. In the assays, the compounds demonstrated cytotoxic activity against tumor cells with different TGI values for the different nuclei studied. In the MCF-7 line, the highest TGI value (13.39 μM) was observed for the CAPA derivative (pyridine), while in our study with the same line, the GI_50_ value was 33.27 μM. It is worth noting that the TGI values found in the study by Santos et al. [7] for the HaCaT line ranged between 1.64 and 6.17 μM across the different nuclei studied.

Meanwhile, the compounds **3c** and **3d** were not active against both breast tumor cell lines used (T47-D and MCF-7). Indole derivative exhibited a GI_50_ of 9.44 μM and in the HaCaT tumor line, a GI_50_ of 4.29 μM. As previously described, some compounds showed proximity in GI_50_ values between tumor and non-tumor cell lines, indicating lower selectivity and, consequently, potential toxicity to healthy cells. However, the results demonstrate that the GI_50_ values of all derivatives were higher than those observed for the reference drug (positive control), *m*-AMSA.

Similar results to our findings were reported by Kim et al. [38], who studied a series of analogs based on the indole nucleus. The results demonstrated that compounds (**3a**–**b** and **3d**–**e**) were more cytotoxic against MCF-7 cells than gefitinib, with GI_50_ values of 19.3, 9.6, 4.6, and 8.3 µM, respectively. Specifically, the inhibitory activity of compound **3d** (GI_50_: 4.6 µM) was approximately 4.5 times stronger than that of gefitinib (GI_50_: 20.8 µM). These results confirm the cytotoxicity of indole compounds such as **3c** studied here, which presented a GI_50_ of 9.44 µM for the MCF-7 cell line.

As previously described, some compounds exhibited similar GI_50_ values between tumor and non-tumor cell lines, which may indicate potential toxicity to healthy cells. However, the results demonstrate that the GI_50_ values of all derivatives were higher than those observed for the reference drug (positive control), m-AMSA, which already has reported toxicity against non-tumor cells and hemolytic undesired effects not reported for **3a-3d** derivatives.

### 2.6. Teratogenic Potential and In Vivo Toxicity

Several anticancer drugs have their use limited due to the lack of safety data regarding toxicity in preclinical phase studies [43]. Such a problem can be addressed through in vivo analyses of the potential toxicity of a drug candidate [57]. For studies on toxicity, teratogenicity, and cardiotoxicity, zebrafish is widely recognized due to its genetic similarity to humans [58]. Despite its popularity, studies on zebrafish embryos with promising nuclei for cancer treatment remain limited. Recently, Santos et al. [7] studied nitrogen heterocyclic derivatives and evaluated the teratogenic and toxicological profiles against zebrafish embryos, observing that the derivatives did not present toxicity or teratogenic activity.

In the study conducted here, zebrafish embryos were exposed to the compounds **3a**, **3b**, **3c**, and **3d** at concentrations of 0.5 μM and 1 μM, with their embryonic development evaluated over 72 h post-fertilization (hpf). The data are presented in Table 9. During the assay, zebrafish embryos at 24 hpf showed 10% embryo coagulation in the negative control (E3 medium) and 100% embryo coagulation for the positive control, 3.4-dichloroaniline. Regarding the exposure to the derivatives, at 24 hpf, 10% coagulation was observed for the compound **3a** (acridine) at 0.5 μM, the same percentage of coagulation as was observed in the negative control. The toxicity of spiroacridine derivatives in zebrafish embryos was reported by Duarte et al. [59]. The authors describe that the compound AMTAC-06 did not induce lethal effects or morphological alterations during embryonic and larval development, with an LC_50_ greater than 126.2 μM after 96 h of exposure, confirming our findings for our acridine derivative (**3a**).

**Figure 8:**
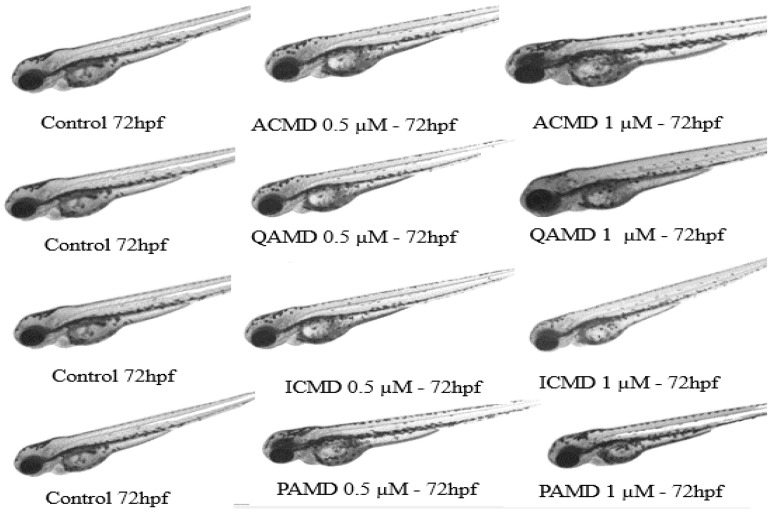
Embryonic development of zebrafish after 72 hpf in contact with the derivatives **3a**, **3b**, **3c**, and **3d** at two concentrations (0.5 and 1 μM).

At 24 hpf, coagulation was also observed in 2.5% and 5% of the embryos exposed to **3b** (quinoline) and **3c** (indole) at 1 μM, respectively. By the end of the 72 hpf exposure to the derivatives, no other embryos exhibited rates of mortality, malformations, or delays in embryonic development. These data indicate the absence of toxicity of the derivatives to the embryos, as the coagulation rate is within the prescribed parameters [60], which predicts a survival rate of 80% after 72 hpf [61,62]. Figure 8 shows the embryonic development at 72 hpf after exposure to the derivatives.

Quinoline derivative (**3b**), in addition to not exhibiting toxicity to the embryos, also demonstrated anticancer activity against the T47-D tumor cell line, diverging from the findings described by Sittaramane et al. [63]. The authors determined the toxicological and anticancer activity of derivatives with a quinoline nucleus, with these compounds inhibiting the development of zebrafish embryos. The most toxic to the embryos were the compounds 2-benzyl-1,1,1-trifluoro-3-(quinolin-2-yl)propan-2-ol (2) and trifluoro-3-(isoquinolin-1-yl)-2-(thiophen-2-yl)propan-2-ol (3), with an LC50 value of 14.14 μM. However, compound (2) possessed potent antiproliferative activity against tumor cells.

The toxicity and teratogenicity of nitrogen heterocyclic derivatives were described in the work of Santos et al. [7], where acridine, quinoline, indole, and pyridine compounds did not present significant mortality rates in embryos, with coagulation rates ranging from 2.5% to 20%, the highest rate (20%) observed for the compound with a pyridine nucleus. This contrasts with our findings for the compound **3d** (pyridine), which did not exhibit any embryo coagulation during the 72 hpf. The authors also reported no tail detachment at 24 hpf for the acridine compound at 0.5 μM and 1 μM, and for the quinoline and indole derivatives, no tail detachment was also evidenced at the same concentrations and exposure interval, a parameter not observed in our study.

In addition to toxicity, cardiotoxicity is an increasing concern in cancer treatment. Chemotherapeutic treatment contributes to deleterious cardiac effects, which may be additive or synergistic [64,65]. Cardiovascular events such as cardiomyopathy, myocardial fibrosis, and arrhythmias are secondary effects associated with oncological treatment [64,66,67]. For example, epirubicin, idarubicin, daunorubicin, and doxorubicin anthracyclines are used in cancer therapy, and the cardiotoxicity of these agents has been reported [68,69]. The treatment of cardiotoxicity in cancer patients requires therapeutic alternatives and ongoing preclinical phase studies to prevent unwanted cardiac effects [68,70]. Therefore, the analysis of cardio-oncology is necessary to prevent or mitigate cardiovascular damage from potential drug candidates.

**Figure 9:**
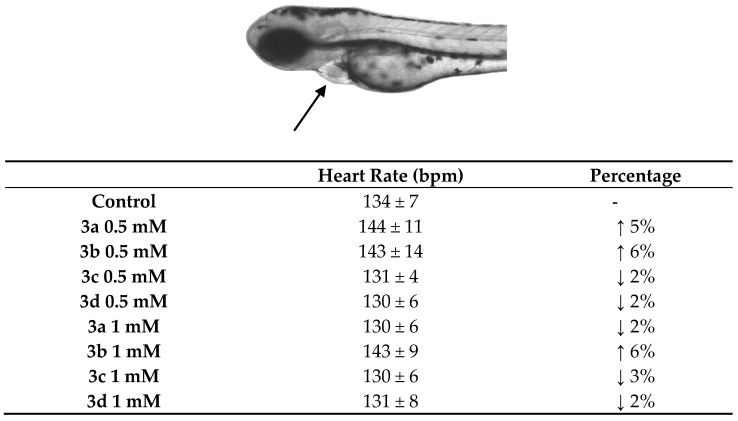
Heart rate of zebrafish embryos. Heartbeats were quantified at 72 hpf, and the experiment was performed in quadruplicate by two researchers. ↑ represents a percentage increase in heart rate and ↓ a decrease compared to the control. Values are presented as means ± SE (*n* = 20).

The heart rate of the embryos was monitored, and both increases and decreases in heart rate were observed at the two tested concentrations, ranging between 130 and 143 bpm, within the expected range for zebrafish, which varies from 120 to 180 bpm [71,72]. Acridine compound (**3a**) at 0.5 μM, compared to the control, increased the heart rate by 5%, without significant statistical differences (Figure 9). Ramachandran and Nagarajan [73] evaluated the toxicity of new acridine–flavone hybrids in zebrafish larvae, in which the compounds did not show cardiac or hepatotoxicity, corroborating our findings.

Quinoline derivative (**3b**) at 0.5 and 1 μM caused a greater increase in heart rate in zebrafish embryos. However, this increase was only 6% compared to the control. Although these data show variations in heartbeats, there are no statistically significant differences, indicating no cardiac toxicity. In contrast to previous studies, Han et al. [74] evaluated the toxicity of 12 different (fluoro)quinolones in zebrafish embryos and reported that all antibiotics at concentrations ranging from 0.1 to 20 mM caused different types of malformations such as head deformation, shortened tail, reduced pigmentation, pericardial edema, cardiac malformation, and death, with more than 80% of the mortality rate associated with cardiac dysfunction. These observations are distinct from the findings for the quinoline studied here.

Teratogenic and toxicological studies were conducted by Santos et al. [7]. In this study, under the same experimental conditions used here, zebrafish embryos exposed to nitrogen heterocyclic derivatives containing indole and pyridine nuclei showed an increase in the heart rate of the embryos. The indole derivative (CAIC) at 0.5 μM increased the heart rate of the embryos by 14.8% compared to the control, and this was statistically different compared to the control (*p* < 0.001). The pyridine compound (CAPA) increased by 11.3% (0.5 μM) and 10.5% (1 μM). One of the mechanisms that may be related to these findings is the generation of reactive oxygen or nitrogen species. Here, indole and pyridine derivatives promoted a decrease in heart rate of 2% to 3%; however, compared to the control, this reduction was not statistically different. Therefore, it is possible to assume that the compounds studied here appear more promising due to having lower mortality rates without statistically significant increases in heart rate, especially in the quinoline derivative, which stood out in all interaction assays.

Finally, another useful biochemical parameter for monitoring embryo toxicity in zebrafish tissues is redox balance enzyme activity evaluation. The generation of reactive oxygen species (ROS) is a physiological process in cellular metabolism [75]. However, an excess of ROS can cause damage to cells, leading to oxidative stress and the onset of diseases, including cardiovascular diseases and cancer [75,76]. The antioxidant defense system includes the following enzymes: superoxide dismutase (SOD), catalase (CAT), and glutathione peroxidase (GPX). These act especially against the superoxide radical anion (*O*_2_^−^) and hydrogen peroxide (*H*_2_*O*_2_) [77].

**Figure 10:**
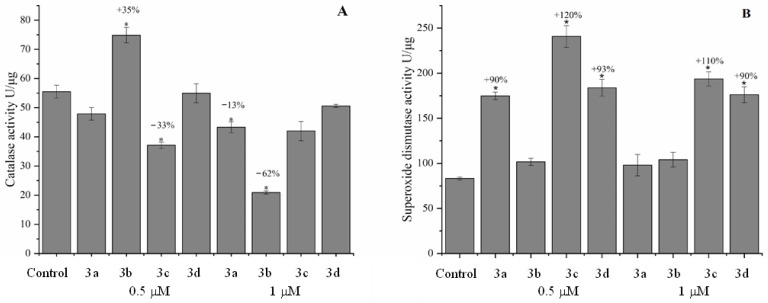
The results represent enzymatic activity (U/μg of protein). The asterisk (*) represents a value of *p* = 0.001. (**A**) catalase activity, * represents a value of *p* < 0.03, and (**B**) represents superoxide dismutase activity. * represents a value of *p* < 0.01. Values are presented as means ± standard error (*n* = 20).

Superoxide dismutase (SOD) and catalase (CAT) are antioxidant enzymes that catalyze the conversion of reactive species into non-reactive products. SOD catalyzes the conversion of the superoxide anion into hydrogen peroxide, and catalase catalyzes the conversion of hydrogen peroxide into oxygen and water [78]. In the present study, the enzymatic activity of CAT showed both an increase and a reduction in all studied nitrogen heterocycles (Figure 10). Acridine derivative (**3a**) reduced enzyme activity at both tested concentrations, with no statistically significant reduction at 0.5 μM. On the other hand, compared to the control, at the concentration of 1 μM, CAT activity was reduced by 13%, with statistical significance. Statistical differences were also observed for **3b** at 0.5 and 1 μM and **3c** at 0.5 μM. Enzyme activity increased by 35% at 0.5 μM and reduced by 62% at 1 μM of **3b**. Compared to the control, **3c** (0.5 μM) reduced CAT enzymatic activity by 33%, which may indicate a deficiency in enzyme activity or that the enzyme’s function is compromised in the presence of the compounds, which can contribute to the emergence of ROS. After all, it has been reported in the literature that the decrease in antioxidant defenses causes the development of diseases such as Alzheimer’s, Parkinson’s, and cancer [79].

In the SOD activity, no heterocyclic compound decreased enzymatic activity. However, an increase in catalytic activity and statistical differences compared to the control were observed. This represents an increase of 90% (**3a** 0.5 μM), 120% (**3c** 0.5 μM), 110% (**3c** 1 μM), 93% (**3d** 0.5 μM), and 90% (**3d** 1 μM). Although **3b** increased the enzymatic activity of SOD, there was no statistical difference (*p* = 0.9). Both SOD and CAT enzymatic activity were altered, except for **3b**, which only altered the enzymatic activity of CAT. The increase in SOD and CAT activities can also be justified by the elevated production of ROS [80].

Considering the increase in heart rate in embryos and changes in the activity of antioxidant enzymes (CAT and SOD), it is possible to hypothesize a potential alteration in the redox state. However, this variation in enzymatic activity can also be considered a cellular response against oxidative stress. Therefore, more information is needed regarding the generation of radicals, considering that in some types of cancer, the downregulation (decrease) of catalase expression can contribute to a more oxidative cellular environment and aid in tumor progression. On the other hand, catalase can be expressed more abundantly in some types of cancer, which may be associated with tumor cell resistance to treatment [81].

## 3. Materials and Methods

### 3.1. Chemicals, Reagents, and Equipment

The starting materials ethyl 2-cyanoacetate and aniline were acquired from Sigma-Aldrich and used for the synthesis of the intermediate reagent 2-cyano-N’-phenylacetamide, as well as the acridine, quinoline, indole, and pyridine aldehydes. Common solvents used for synthesis and analysis were provided by Sigma-Aldrich (Saint Louis, MO, USA), Merck (Darmstadt, Germany), and Fluka (Buchs, SG, Switzerland) and used without purification. Melting points were measured in capillary tubes using a Quimis Model 340 apparatus (Quimis, Diadema, Brazil). Thin-layer chromatography (TLC) was performed on Fluka Analytical silica gel 60 plates with a thickness of 0.25 mm and fluorescent indicator 254 nm and visualized under UV light (254 or 365 nm).

Infrared spectra were obtained using KBr disks on an IRPrestige-21 spectrometer, Shimadzu^®^ (Kyoto, Japan). Data were processed using Origin software 8.0. Proton nuclear magnetic resonance (1H-NMR) and carbon-13 NMR experiments were conducted on a Varian Model Plus spectrophotometer (Varian, Santa Clara, CA, USA) operating at 300 MHz, using DMSO-d6 as the solvent. Spectra were plotted and interpreted using Mestre Nova 12.0 software. Chemical shifts (*δ*) were expressed in ppm, and coupling constants (*J*) were given in Hertz (Hz). Signal multiplicities were designated as follows: singlet (*s*), doublet (*d*), multiplet (*m*). Mass spectra were recorded using matrix-assisted laser desorption/ionization time-of-flight (MALDI-TOF) on an Autoflex III mass spectrometer (Bruker Daltonics, Billerica, MA, USA).

The Salmon sperm DNA (ssDNA) and ethidium bromide (EB) used for interaction analysis were procured from Sigma-Aldrich (Saint Louis, MO, USA). Human serum albumin (HSA) utilized in this study was obtained following the method described by Alves et al. [10]. The compounds were prepared as solutions in dimethyl sulfoxide (DMSO), followed by further dilution in Tris-HCl buffer (0.1 M; pH 7.6). UV-visible absorption spectra and fluorescent emission spectra were recorded using the Thermo Scientific 51119200 spectrometer and the JASCO FP-6300 spectrofluorometer (Tokyo, Japan), respectively.

### 3.2. Procedure for Synthesis of 2-Cyano-N-Phenyl Acrylamide Derivatives

The intermediate 2-cyano-N-phenylacetamide (2) was obtained through a nucleophilic addition reaction between ethyl 2-cyanoacetate (1) and aniline. The final isopropyl acrylamides (**3a**–**3d**) were obtained through a nucleophilic addition reaction between 2-cyano-N-phenylacrylamide (2) and different aromatic aldehydes in an ethanolic and basic medium at a temperature of 78 °C, with the reaction time varying from 3 to 96 h, followed by the spontaneous cyclization of **3a** according to Scheme 1. The reaction was monitored by Analytical Thin-Layer Chromatography (TLC), determining the end of the reaction. The reaction was filtered, and the obtained crystals were washed with cold distilled water and then recrystallized in ethanol. The ^1^H and ^13^C NMR, IR spectroscopy, and mass spectrometry data are presented in Supplementary Material Figures S1–S14.

#### 3.2.1. (*E*)5′-Oxo-1′-Phenyl-1′,5′-Dihydro-10*H*-Spiro[Acridine-9,2′-Pyrrole]-4′-Carbonitrile (ACMD)—Compound (**3a**)

Yellow powder. Formula: C_23_H_15_N_3_O; M.W.: 349.1215 g/mol; Yield: 75%; Melting Point: 240–242 °C; R*f*: 0.57 (*n*-hexane/EtOAc 6:4). IR (KBr, cm^−1^): 3314 (NH), 3094 (CH), 2235 (CN), 1695 (C=O), 1450 and 1343 (aromatic CN). 1H NMR (DMSO-*d*6): *δ* 9.63 (*s*, 1H, NH), 8.57 (*s*, 1H, CH), 7.22 (*m*, 2H, *J* = 8.4 Hz e *J* = 1.6 Hz), 7.12 (*m*, 3H, aromatic), 7.07 (*m*, 2H, spiroacridine), 6.85 (*m*, 4H, spiroacridine), 6.76 (*m*, 2H, spiroacridine). 13C NMR: *δ* 70.6, 108.9, 112.2, 113.1, 115.5, 120.6, 125.4, 127.1, 128.1, 129.0, 130.5, 136.2, 138.7, 163.3, 164.1. HRMS *m*/*z* [*M*+H]+: calculated: 349.3847; found: 350.1168.

#### 3.2.2. (*E*)-2-Cyano-*N*-Phenyl-3-(Quinoline-4-yl)Acrylamide (QAMD)—Compound (**3b**)

Yellow powder. Formula: C_19_H_13_N_3_O; M.W.: 299.1059 g/mol; Yield: 55%; Melting Point: 195–197 °C; R*f*: 0.50 (*n*-hexane/EtOAc 1:1). IR (KBr, cm^−1^): 3470 (NH), 3262 (CH), 2233 (CN), 1681 (C=O), 1550 and 1498 (aromatic C=C). 1H NMR (DMSO-*d*6): *δ* 10.34 (*s*, 1H, NH), 9.04 (*d*, 1H, *J* = 4.4 Hz aromatic quinoline), 8.24 (*d*, 1H, *J* = 8.0 Hz aromatic quinoline), 8.16 (*d*, 1H, *J* = 8.4 Hz aromatic quinoline), 7.89 (*t*, 1H, *J* = 8.0 Hz, aromatic), 7.75 (*m*, 3H, aromatic), 7.54 (*d*, 1H, *J* = 4.4 Hz, aromatic quinoline), 7.39 (*t*, 2H, *J* = 7.6 Hz aromatic), 7.17 (*t*, 1H, *J* = 7.6 Hz aromatic quinoline), 5.73 (*s*, 1H, C=CH). 13C NMR: *δ* 114.4, 118.2, 121.4, 124.0, 125.0, 125.5, 128.2, 129.2, 130.2, 130.8, 137.5, 137.7, 147.8, 150.9, 160.0. HRMS *m*/*z* [*M*+H]+: calculated: 299.1059; found: 300.1570.

#### 3.2.3. (*E*)-2-Cyano-3-(1*H*-Indol-3-yl)-*N*-Phenylacrylamide (ICMD)—Compound (**3c**)

Yellow powder. Formula: C_18_H_13_N_3_O; M.W.: 287.1059 g/mol; Yield: 60%; Melting Point: 276–277 °C; R*f*: 0.55 (*n*-hexane/EtOAc 7:3). IR (KBr, cm^−1^): 3322 (NH), 3040 (CH), 2212 (CN), 1640 (C=O), 1570 and 1442 (aromatic C=C). 1H NMR (DMSO-*d*6): *δ* 12.43 (*d*, 1H, NH, indole), 10.17 (*s*, 1H, NH, amide), 8.62 (*s*, 1H, *C=CH*), 8.55 (*s*, 1H, *C=CH,* indole), 8.01 (*m*, 1H, aromatic indole), 7.70 (*d*, 2H, *J* = 7.9 Hz, aromatic), 7.59 (*m*, 1H, aromatic), 7.37 (*t*, 2H, *J* = 7.8 Hz, aromatic), 7.29 (*m*, 2H, aromatic), 7.13 (*t*, 1H, *J* = 7.4 Hz aromatic). ^13^C NMR: *δ* 110.3, 113.2, 119.1, 121.1, 121.1, 123.8, 124.4, 128.1, 129.1, 131.1, 137.0, 140.4, 143.2, 161.7. HRMS *m*/*z* [*M*+H]+: calculated: 287.1059; found: 310.0880.

#### 3.2.4. (*E*)-2-Cyano-*N*-Phenyl-3-(Pyridin-4-yl)Acrylamide (PAMD)—Compound (**3d**)

Yellow powder. Formula: C_15_H_11_N_3_O; M.W.: 249.0902 g/mol; Yield: 32%; Melting Point: 220–222 °C; R*f*: 0.40 (*n*-hexane/EtOAc 1:1). IR (KBr, cm^−1^): 3330 (NH), 3019 (CH), 2225 (CN), 1676 (C=O), 1598 and 1444 (aromatic C=C). 1H NMR (DMSO-*d*6): *δ* 10.54 (*s*, 1H, NH), 8.81 (*s*, 2H, aromatic pyridine), 8.27 (*s*, 1H, *C=CH*), 7.81 (*d*, 2H, *J* = 5.2 Hz, aromatic pyridine), 7.66 (*d*, 2H, *J* = 7.6 Hz, aromatic), 7.37 (*t*, 2H, *J* = 8.0 Hz, aromatic), 7.14 (*t*, 1H, *J* = 7.6 Hz, aromatic). ^13^C NMR: *δ* 112.5, 115.7, 121.0, 123.4, 125.1, 129.3, 138.4, 139.5, 148.8, 151.2, 160.1. HRMS *m*/*z* [*M*+H]+: calculated: 249.0902; found: 250.1020.

### 3.3. UV–Vis Absorption Spectroscopy Studies with DNA

Initially, the ssDNA solution was dissolved in Tris-HCl buffer (0.1 M, pH 7.6) and stored at 8 °C for 24 h. The purity of the ssDNA solution was determined using an absorption spectroscopy assay at wavelengths of 260 nm and 280 nm. DMSO was used for compound dilution, with a final concentration of 1 mM. The test solutions of the compounds **3a**, **3b**, **3c,** and **3d** were prepared at a concentration of 5 μM. These solutions were exposed to increasing concentrations of ssDNA (10, 20, 40, 60, 80, and 100 μM). The test solutions (ssDNA and compounds) were homogenized and left to rest at 25 °C for 10 min. The absorbance readings of the samples were then performed using a UV-vis Thermo Scientific 51119200 spectrophotometer in 96-well plates, at a wavelength range of 200–600 nm. The McGhee and Von Hippel [82] equation:

[Derivative]/(εa − εf) = [Derivative]/(εb − εf) + 1/Kb (εb − εf) [DNA]/(εa − εf)

[DNA]/(εb − εf) + 1/Kb (εb − εf) where εa, εb, and εf are the apparent, bound, and free extinction coefficients, respectively. The graphs for [Compound]/( εa − εf) versus [Compound] and [DNA]/(εa − εf) versus [DNA] were used to obtain the Kb values from the ratio of inclination and interception, using the software SigmaPlot 10.0, which was used to determine the binding constant (Kb). The analyses were performed in triplicate.

#### Fluorescent DNA Probe Assay

The fluorescence emissions of EB were measured in the presence and absence of ssDNA. The investigation involved a concentration of 10 μM of EB and 100 μM of ssDNA. These measurements were conducted using a rectangular quartz cuvette with a 1 cm optical path and a JASCO FP-6300 spectrofluorometer (Tokyo, Japan). For the EB fluorescence spectra, excitation was set at 526 nm, and emission was recorded in the range of 550 to 100 nm. To evaluate the displacement of EB by the tested compounds, solutions containing EB-ssDNA were exposed to the derivatives at concentrations of 10, 20, 40, 60, 80, and 100 μM in Tris-HCl buffer (0.1 M, pH 7.6). The tests were conducted in triplicate. All solutions were carefully mixed and allowed to stabilize for 10 min in the mentioned buffer before undergoing fluorescence analysis. The concentrations used in the test solutions were determined after a scan of the absorption profile of the free test compounds was conducted. The emission suppression behavior was analyzed using the Stern–Volmer equation (Ksv) [83]:

F0/F = 1 + Ksv [Q]

where F0 and F are the fluorescence intensities in the absence and in the presence of the derivatives, respectively. Ksv is the constant of linear suppression. [Q] represents the concentration of the compound. Binding data were obtained with the software SigmaPlot 10.0.

### 3.4. Physicochemical and Pharmacokinetic Predictions

The MarvinSketch^®^ academic license platform (https://chemaxon.com/products/marvin, accessed on 9 December 2025) was used for the two-dimensional representation of the chemical structure and to quantitatively estimate the similarity to drugs based on multiple parameter optimization (MPO), following the equation:

$$D=\sum_{i=1}^{m}w_{K}T_{k}(x_{k}^{0})$$

where w is the weighting factor assigned to the calculated value x of a property k, which varies from 0 to 1, staying within (xₖ ≤ xₐ) or outside (x_b_ < xₖ) the desirability limit.

The MPO estimate covers parameters such as intrinsic lipophilicity (logP ≤ 3), pH (logD ≤ 2), molecular weight (200 < MW ≤ 500 g/mol), topological polar surface area (40 < TPSA ≤ 90 Å^2^), number of hydrogen bond donors (HBD ≤ 1), and pKa (≤8), which will result in a score ranging from 0 to 6 (M = 6), which is a pharmacokinetic feasibility score. In addition, drug similarity criteria from Lipinski’s “rule of five” were also applied, and compatibility with the rules of Veber, Ghoshe, Egan, and Muegge was assessed using the SwissADME platform (http://www.swissadme.ch/, accessed on 9 December 2025).

In order to understand parameters related to physicochemical and pharmacokinetic aspects, different platforms were used, such as SwissADME and ADMETlab 3.0 (https://admetlab3.scbdd.com/, accessed on 10 December 2025) to predict factors related to absorption (Caco-2 permeability -2 and MDCK permeability), distribution (plasma protein binding, distribution volume, and blood–brain barrier permeability), metabolism (CYP1A2, CYP2C19, and CYP3A4), and excretion (clearance). The half-life was determined using the following formula:

t_1/2_ = (0.693 × V_d_) ÷ Cl

In addition, other platforms such as Xenosite (https://xenosite.org/) were used to describe metabolization points. Finally, aspects related to the toxicity of the compounds were described using ProTox-3.0 (https://tox.charite.de/, accessed on 11 December 2025) and TopTox (https://stoptox.mml.unc.edu/, accessed on 11 December 2025).

### 3.5. Molecular Docking

AutoDock 4.2.6, combined with the Lamarckian genetic algorithm [84], was used for the molecular docking analyses.

#### 3.5.1. Ligand Structure Preparation

The ChemDraw 15.0 software was used to construct the structure of the analyzed compounds, and the structures were optimized based on the MM2 semi-empirical theory. After optimization, the structures were saved in pdb format for docking studies.

#### 3.5.2. Topoisomerase IIα and HSA in the Presence of Ligands

For topoisomerase IIα, the parameters for generating the three-dimensional grid (3D grids) were previously configured in AutoGrid 4.2.6. This intercalating agent contained 70 × 70 × 70 grid points with a spacing of 0.0375 nm. The grid was centered at the position of the co-crystallized ligand in the A chain of the protein. For HSA, the 3D grids were configured using AutoDockTools 4.2.6 and AutoGrid 4.2.6. In both cases, the 3D grids had dimensions of 126 × 126 × 126 points with a spacing of 0.0375 nm. The center was defined as the first grid at the position of the co-crystallized ligand in site I of the A chain of the protein, while the second grid was centered at the position of the co-crystallized ligand in site II of the A chain of the protein. For both macromolecules, the Lamarckian genetic algorithm in AutoDock 4.2.6 was applied to search for the optimal conformation and orientation of the ligands. From the docking experiments, 100 conformations were generated and analyzed. The lowest energy conformation for each ligand was determined and examined using AutoDockTools and Discovery Studio Visualizer [7].

### 3.6. Human Topoisomerase IIα Inhibition

The topoisomerase IIα inhibition assay was conducted following the methodology described by Almeida et al. [8]. Subsequently, quantitative analysis was performed by densitometry to obtain the percentage of enzymatic inhibition by the derivatives, using image processing software (Scion Image, Beta 4.0.2), according to Gouveia et al. [20].

### 3.7. Evaluation of Antiproliferative Activity In Vitro

The sulforhodamine B method, as described by Monks et al. [85], was used in the antiproliferative assay with tumor and non-tumor cells kindly supplied by the cell bank from Faculdade de Ciências Farmacêuticas, Universidade Estadual de Campinas (UNICAMP), Campinas-SP, Brazil, initially constituted by National Cancer Institute (NCI) cell lines under Professor João Ernesto de Carvalho’s administration. The breast cancer cell lines MCF-7 and T47D, as well as immortalized normal human keratinocytes (HaCaT), were utilized. In cell culture, the cells were grown and maintained in RPMI-1640 medium supplemented with 5% fetal bovine serum and 1% antibiotics, consisting of penicillin (1000 IU/mL) and streptomycin (1000 μg/mL). For the assay, 6 × 10^3^ cells were plated in 96-well plates and incubated at 37 °C, 5% CO_2_ for 24 h. After this period, the cells were incubated with the compounds **3a**, **3b**, **3c**, and **3d** at concentrations of 0.25, 2.5, 25, and 250 μM, which were previously dissolved in DMSO and diluted in RPMI-1640 medium. The same concentrations were used for the positive control, amsacrine (m-AMSA). After 48 h of incubation and exposure to the compounds, the cells were fixed with 50% trichloroacetic acid solution. Cell proliferation was quantified by absorbance reading at a wavelength of 540 nm, using sulforhodamine B stain. Non-linear regression analysis with Origin 8.0^®^ software (OriginLab Corporation, Northampton, MA, USA) was used to determine the concentration values that inhibited 50% of cell growth (GI_50_) for each cell line. Each experimental assay was performed in triplicate.

### 3.8. Hemolytic Activity Studies of the Derivatives

The human erythrocytes used were obtained from a voluntary donation by a healthy donor, with a volume of 5 mL. The protocol used was approved by the Ethics Committee (protocol number 4.733.296, on 5 August 2020) following the Resolution CNS 466/12, which regulates research involving human beings in Brazil. The collected blood sample was subjected to 3 washes with saline solution (0.9% NaCl) and centrifuged at 2500 rpm for 5 min. All plasma residue was removed from the sample, resulting in a concentrated red blood cell solution. The 0.5% stock solution was prepared by diluting 250 μL of the concentrated solution in 50 mL of saline solution. In the test solution, 2 mL of the red blood cell stock solution was used. For the assay, the compounds **3a**, **3b**, **3c,** and **3d** were previously diluted in 1% DMSO, with a final concentration of 1 mM, and then test solutions were prepared. The red blood cell test solution was exposed to concentrations of 5, 10, 25, 50, and 100 μM of the compounds. The negative control used was 2 mL of red blood cells mixed with 10 μL of saline solution, while the positive control consisted of 2 mL of red blood cells supplemented with 10 μL of 1% Triton X-100. The negative control corresponded to 0% hemolysis and the positive control to 100% hemolysis. All samples were incubated for one hour at room temperature and then centrifuged at 2500 rpm for 5 min at 4 °C. The supernatant was collected and transferred to a 96-well plate for reading on a spectrophotometer at a wavelength of 540 nm. The entire assay was conducted in triplicate. Equation: % Hemolysis = [(_Sample_ Abs − _Negative Control_ Abs)/_Positive Control_ Absorbance)] × 100, where _Sample_ Abs is the absorbance of the test solution (Red Blood Cells + Derivatives), _Negative Control_ Abs is the absorbance of the negative control (Red Blood Cells + Saline Solution), and _Positive Control_ Abs is the absorbance of the positive control (Red Blood Cells + Triton), was used to calculate the hemolysis rate [86].

### 3.9. In Vivo Studies

#### 3.9.1. Care and Obtention of Zebrafish Embryos

Zebrafish (*Danio rerio*) wild type was commercially obtained and maintained in a laboratory at the University of Pernambuco (UPE), Garanhuns Campus. The adult fish (over one year old) were separated into male and female groups in 16 L tanks connected to a water recirculation and filtration system, with specific and appropriate conditions for the reproductive cycle, including a light/dark cycle of 14 h of light and 10 h of darkness, pH of 7.2, and a temperature of 26 °C. The fish were fed daily ad libitum with Tropical^®^ fish food (Poytara) and *Artemia* spp. Only healthy animals were used in reproductive procedures. Animal health was assessed by behavioral observation, appropriate gill coloration, and adequate reproductive capacity. All animal care, including humane endpoints were conducted according to ethical standards and approved by the Ethics Committee on the Use of Animals of the University of Pernambuco (CEUA-UPE) under Process No. 008/2021—approved on 21 September 2021, following the guidelines of CONCEA (Brazilian National Council for the Control of Animal Experimentation).

After 2 months of acclimatization to laboratory conditions, the fish were subjected to a reproductive event to obtain embryos, using one female and two males. The fish were separated and transferred to a breeding tank with a gridded bottom at around 4:00 PM while maintaining the previously described conditions. The reproduction occurred in the early hours of the following day, and the embryos were collected and analyzed for viability using a Motic Phantera microscope. Embryos considered non-viable (coagulated or unfertilized) were discarded, and viable embryos were used for exposure.

#### 3.9.2. Teratogenic Potential

The teratogenic studies followed the guidelines established by the OECD [60], including the sample size. After analyzing the viable embryos following the guidelines, the fertilized eggs were washed with E3 medium (composed of 5 mM NaCl, 0.17 mM KCl, 0.33 mM MgSO_4_ · 7H_2_O, and 0.33 mM CaCl_2_ at pH 7.2) and then incubated at 25 °C. In 24-well plates, 20 embryos per well (in duplicate) were exposed to the compounds **3a, 3b, 3c,** and **3d** at concentrations of 1 μM and 0.5 μM, starting 6 h post-fertilization (hpf), at a temperature of 26 ± 1 °C. Each well was considered as an experimental unit (*n* = 20) and the animals were randomly assigned to the wells. The positive control used was 3,4-dichloroaniline, and the negative control was E3 medium. The group allocation was determined by the independent researchers as a blinding strategy. The embryos were monitored at 24 hpf, 48 hpf, and 72 hpf, considering lethality parameters such as coagulated embryos, absence of somite formation, failure of tail detachment, and absence of heartbeat [60]. During the 72 hpf, the embryos were photographed using a Motic Phantera microscope. A total of 200 embryos were employed in this study.

#### 3.9.3. Enzymatic Assays of Embryo Tissues

After 72 hpf of embryo exposure to the derivatives, enzymatic studies were conducted on the enzymes superoxide dismutase (SOD) (EC 1.15.1.1) and catalase (CAT) (EC 1.11.1.6). For tissue homogenization, a tissue homogenizer (NT-136—Nova Técnica) was used, along with 100 μL of ice-cold PBS buffer at 0.1 M, pH 7.2, supplemented with sodium orthovanadate (1 mM) and phenylmethylsulfonyl fluoride (PMSF, 200 μg/mL). After homogenization, this solution was centrifuged for 10 min at 800× *g* (4 °C) in a Thermo Scientific—Heraeus megafuge 16R; the pellet was discarded, and the supernatant was used for the enzymatic assays. Protein quantification was previously determined using the bicinchoninic acid method [87]. Following Misra and Fridovich [88], adaptations were made, and the following conditions were established for SOD activity evaluation at 480 nm: epinephrine (60 mM) diluted in 0.05% acetic acid was used as a substrate, monitored for 9 min in the presence of 50 mM glycine buffer at pH 10.0. CAT activity was evaluated at 240 nm with 300 mM H2O2 as the substrate. The catalytic event was monitored for 3 min [89]. All assays were performed in triplicate.

#### 3.9.4. Statistical Analysis

The data obtained from the assays were initially subjected to the Kolmogorov–Smirnov test to assess normality. Subsequently, one-way ANOVA followed by Tukey’s post hoc test was performed. Results with *p* < 0.05 were considered statistically significant. Statistical analyses were conducted using IBM^®^ SPSS^®^ software (Statistics Premium Grad Pack 25 version), and graphs were generated using OriginLab 8^®^.

## 4. Conclusions

Four derivatives were synthetized and their chemical structures were confirmed: **3a** (spiroacridine), **3b** (quinoline), **3c** (indole), and **3d** (pyridine). The biological activity of the four molecules **3a**–**3d** indicates their ability to interact with target biomolecules in cancer therapy. Among the analyzed compounds **3a**–**3d**, those that exhibited the most significant interaction with DNA, as assessed by UV-vis spectroscopy and fluorescence probes, were **3a** (acridine) and **3b** (quinoline), with Kb values of 2.23 × 10^5^ and 1.41 × 10^5^, and Ksv values of 0.67 × 10^3^ and 0.63 × 10^3^ (EB), respectively. In the molecular docking study, **3b** demonstrated the highest binding energy. All compounds inhibited topoisomerase activity and were cytotoxic against tumor cells while exhibiting minimal hemolytic activity, suggesting potential efficacy in cancer therapy. In the evaluation of teratogenicity, toxicity, and cardiotoxicity in zebrafish embryos, no toxic effects were observed at the tested concentrations. However, further investigations are necessary to assess their influence on redox balance, considering the observed changes in superoxide dismutase (SOD) and catalase (CAT) activity. Thus, based on the evidence presented in this study, the employed techniques demonstrate the value of this new class of nitrogen heterocycles and provide insights into the development of novel anticancer molecules. Reducing the acridine nucleus to a quinoline core while maintaining the N-phenyl-substituted amide side group appears to be a promising approach.

## Figures and Tables

**Table 1:** General physicochemical properties of compounds, characteristics of Lipinski, Ghose, Veber, Egan, and Muegge druglikeness rules according to SwissADME and MPO of **3a**, **3b**, **3c**, and **3d** compounds.

Properties	3a	3b	3c	3d
LogP	4.03	3.54	3.48	2.16
LogD	4.03	3.54	3.48	2.16
MW	348.49 g/mol	299.33 g/mol	287.32 g/mol	249.27 g/mol
TPSA	56.13 Å^2^	64.98 Å^2^	65.25 Å^2^	65.25 Å^2^
pKa	−1.22	4.59	−4.30	4.72
HBD	1.00	1.00	2.00	1.00
HBA	3.0	3.0	2.0	3.0
RB	1.0	4.0	4.0	4.0
MR	110.26	90.22	86.78	72.72
CF	C_23_H_15_N_3_O	C_19_H_13_N_3_O	C_18_H_13_N_3_O	C_15_H_11_N_3_O
Criteria				
Lipinski	Yes	Yes	Yes	Yes
Ghose	Yes	Yes	Yes	Yes
Veber	Yes	Yes	Yes	Yes
Egan	Yes	Yes	Yes	Yes
Muegge	Yes	Yes	Yes	Yes
MPO	4.24	4.71	4.52	4.67

**Table 2:** General pharmacokinetic parameters obtained for the compounds **3a**, **3b**, **3c**, and **3d**.

Parameters	3a	3b	3c	3d	Etoposide
Permeability Caco-2	6.60 × 10^−5^ cm/s	1.11 × 10^−5^ cm/s	1.15 × 10^−5^ cm/s	8.57 × 10^−6^ cm/s	9.31 × 10^−7^ cm/s
Permeability MDCK	2.23 × 10^−5^ cm/s	1.48 × 10^−5^ cm/s	1.45 × 10^−5^ cm/s	1.72 × 10^−5^ cm/s	4.66 × 10^−6^ cm/s
Plasma protein binding	98.25%	98.55%	98.65%	95.46%	95.03%
Distribution volume	1.18 L/kg	0.59 L/kg	0.50 L/kg	0.56 L/kg	0.23 L/kg
Permeability to the blood–brain barrier	0%	0.1%	0.2%	0.7%	0.8%
Plasma clearance	6.041 L/h/kg	0.2772 L/h/kg	0.30972 L/h/kg	0.30222 L/h/kg	0.14934 L/h/kg
Half-life	3 h 23 min	1 h 29 min	1 h 15 min	1 h 09 min	1 h 04 min

**Table 3:** Probability results of compounds being enzyme targets for CYP1A2, CYP2C19, and CYP3A4.

Target	Probability			
	3a	3b	3c	3d
CYP1A2	82.1%	3.4%	63.2%	97.9%
CYP2C19	100%	0%	0%	0.4%
CYP3A4	100%	7%	24.3%	59.5

**Table 4:** Prediction and probability of toxicity for the compounds **3a**, **3b**, **3c**, and **3d**.

Target	Prediction and Probability				
	3a	3b	3c	3d	Etoposide
Hepatotoxicity	Active (57%)	Active (57%)	Active (51%)	Active (57%)	Inactive (84%)
Nephrotoxicity	Inactive (76%)	Inactive (71%)	Inactive (64%)	Inactive (71%)	Active (66%)
Cardiotoxicity	Active (92%)	Inactive (83%)	Inactive (84%)	Inactive (81%)	Active (70%)
Carcinogenicity	Inactive (56%)	Inactive (62%)	Inactive (52%)	Inactive (65%)	Inactive (64%)
Mutagenicity	Inactive (52%)	Inactive (64%)	Inactive (56%)	Inactive (61%)	Inactive (85%)
Cytotoxicity	Inactive (80%)	Inactive (80%)	Inactive (71%)	Inactive (80%)	Inactive (90%)

The color green represents inactive toxicity and the color red represents active toxicity according to a probability score ranging from 0 to 1. Color code: Inactive (Probability ≥ 0.7) = green; Inactive (Probability < 0.7) = light green; Active (Probability ≥ 0.7) = red; Active (Probability < 0.7) = light red.

**Table 5:** UV-vis absorption data of derivatives in the presence and absence of ssDNA.

Compound	Ring	λ_max_ Free (nm) ^a^	λ_max_ Bond (nm)	Δλ (nm)	Hyper. (%) ^b^	Hypochr. (%) ^c^	K_b_ ^d^ M^−1^
**3a**	Acridine	363	404	71	-	58.67	2.23 × 10^5^
**3b**	Quinoline	326	404	78	-	91.63	1.41 × 10^5^
**3c**	Indole	390	400	10	54.81	-	5.26 × 10^4^
**3d**	Pyridine	272	269	3	-	37.12	6.46 × 10^4^

^a^ Absorption of ssDNA scanned in the range of 200 and 600 nm. ^b^ Hyperchromicity for complexes formed, derivatives, and 100 mM of ssDNA in comparison to free compounds. ^c^ Hypochromicity for complexes formed, derivatives, and 100 mM of ssDNA in comparison to free compounds. ^d^ Binding constant (Kb) obtained from complexes formed with derivatives-ssDNA.

**Table 6:** Fluorescent emission data of EB-ssDNA complexes in the absence and the presence of compounds.

Compound/Ring	EB-ssDNA			
	λ_máx_ Free (nm) ^a^	λ_máx_ Bound (nm) ^a^	Hypochr. (%) ^b^	Ksv (M^−1^) ^c^
**3a**/Acridine	599	599	59.64	0.67 × 10^3^
**3b**/Quinoline	599	600	73.14	0.63 × 10^3^
**3c**/Indole	599	600	53.29	0.53 × 10^3^
**3d**/Pyridine	599	600	69.72	0.58 × 10^3^

^a^ Data regarding the emissive profile of the EB-ssDNA complex in the presence of different concentrations of the test compounds (10–100 µM). ^b^ Hipochromism resulting from the formation of the interaction between derivatives with the EB-ssDNA. ^c^ Stern–Volmer suppression constant (Ksv) obtained based on fluorescence data with ssDNA.

**Table 7:** Data obtained for the derivatives with the targets 1G3X—Intercalation of a 9Acridine-peptide drug in a DNA dodecamer, 1BNA—Structure of a B-DNA dodecamer: Conformation and dynamics, 5GWK—Human Topoisomerase IIα in complex with DNA and etoposide by molecular docking.

Compound	Binding Energy (kcal/mol) Target: 1G3X	Binding Energy (kcal/mol) Target: 1BNA-	Binding Energy (kcal/mol) Target: 5GWK	Type of Bond	Residues
Ligand 9Acridine	−6.99	-	-	-	-
Ligand Etoposide	-	-	−11.2	Hydrogen boding; π -Alkyl; stacking π- π; Van der Waals;	MET^763^, PRO^803^, ASP^463^, ARG^487^, G^13-D^, G^10-F^ C^8-E^, GLY^488^; GLY^462^
**3a**	−8.07	−6.94	−8.52	Hydrogen boding; π -Alkyl; stacking π- π; π-Cation; π-Anion	ALA^614^; ASP^108^; LYS ^190-466^; ARG^787^; HIS^146^
**3b**	−9.23	−10.37	−8.84	Hydrogen bonding; stacking π- π	ARG^197^; TYR^148^; CYS^200-246^; LEU^250^; ALA^151^; G^13-D^, C^8-E^, A^12-D^, ARG ^487^
**3c**	−8.75	−10.16	−8.76	Hydrogen boding; π-Cation; π-Sigma; π-Sulfur	ALA^26-151^; LYS^106^; GLN^29^; ARG^487^; PHE^149^; TYR^148^; CYS^245-200-246^; LEU^250^; T^9-F^; A^12D^
**3d**	−7.35	−8.65	−7.2	Hydrogen boding π-Cation; π-Sigma; π-Sulfur; π -Alkyl	ALA^151-26^; LYS^106^; ARG^487^; TYR^150^; CYS^200-246^; LEU^250^; GLY^248^

A, C, G and T = Nitrogenous bases Adenine, Cytosine, Guanine, and Thymine, respectively. D; E = DNA strands segments. ARG; ASP; TYR; SER; GLU; LYS; GLY; MET; PRO; ALA; HIS; CYS; LEU; GLN; PHE = Amino acid residues—Topoisomerase IIα enzyme and DNA.

**Table 8:** GI_50_ values in µM for the compounds **3a**, **3b**, **3c**, and **3d** tested in the breast cancer cell lines MCF-7, T47-D, and the non-tumoral cell line HaCaT. Percentage of hemolysis of the derivatives in human erythrocytes.

Derivative	GI_50_ (µM) by Cell Lines ^a^			% Hemolysis in Human Erythrocytes ^d^
	MCF-7	T47-D	HaCaT	
*m*-AMSA ^b^	3.27	0.14	1.44	19%
**3a**	34.41	3.42	>100	6.66%
**3b**	33.62	8.07	2.98	3.08%
**3c**	9.44	n.a	4.29	4.14%
**3d**	33.27	n.a	2.96	2.05%

^a^ MCF-7 (breast adenocarcinoma); T47-D (metastatic cells from an infiltrating ductal carcinoma) and HaCaT (immortalized human keratinocyte). Cell lines were exposed to the compounds in DMSO/RPMI/FBS 5% at 37 °C, 5% CO_2_, for 48 h. ^b^ *m*-AMSA positive controls. Triton X-100 positive controls (100%) and *m*-AMSA ^d^, n.a = Inactive at the highest concentration.

**Table 9:** Zebrafish Embryo Acute Test. The acute test was performed following the Fish Embryo Toxicity (FET) Test of OECD [60] with modifications. Zebrafish embryos are exposed before 90 min post fertilization. The E3 medium was used as a negative control, and 4 mg.L^−1^ of 3,4-dichloroaniline was used as a positive control. The experiment is performed in duplicate (*n* = 20).

Derivative	Lethality Parameters [60]	Exposure Time (h)/Concentration/Percentage (%) of Embryos Within Lethality Parameters [60]		
		24 h	48 h	72 h
Negative Control	Embryo coagulation (%)	10%	0%	0%
	Lack of somite formation (%)	0%	0%	0%
	No tail detachment (%)	0%	0%	0%
	Absence of heartbeat (%)	0%	0%	0%
3,4-dichloroaniline	Embryo coagulation (%)	100%	-	-
		24 h/0.5 and 1 µM	48 h/0.5 and 1 µM	72 h/0.5 and 1 µM
**3a**	Embryo coagulation (%)	10% and 0%	0% and %	0% and 0%
	Lack of somite formation (%)	0%	0%	0%
	No tail detachment (%)	0%	0%	0%
	Absence of heartbeat (%)	0%	0%	0%
**3b**	Embryo coagulation (%)	0% and 2.5%	0%	0%
	Lack of somite formation (%)	0%	0%	0%
	No tail detachment (%)	0%	0%	0%
	Absence of heartbeat (%)	0%	0%	0%
**3c**	Embryo coagulation (%)	0% and 5%	0%	0%
	Lack of somite formation (%)	0%	0%	0%
	No tail detachment (%)	0%	0%	0%
	Absence of heartbeat (%)	0%	0%	0%
**3d**	Embryo coagulation (%)	0%	0%	0%
	Lack of somite formation (%)	0%	0%	0%
	No tail detachment (%)	0%	0%	0%
	Absence of heartbeat (%)	0%	0%	0%

## Data Availability

The original contributions presented in this study are included in the article/Supplementary Material. Further inquiries can be directed to the corresponding author.

## Supplementary Materials

The following supporting information can be downloaded at: https://www.mdpi.com/article/10.3390/ph19030405/s1.
