# Supplementary material for: Biological and Teratogenic Evaluations of Nitrogen Heterocycles for Anticancer Therapy
Source: Pharmaceuticals (Basel). 2026 Mar 1;19(3):405. doi: 10.3390/ph19030405 (PMC13028858; doi:10.3390/ph19030405)
Supplement: Supplementary file 1 [file pharmaceuticals-19-00405-s001.zip › pharmaceuticals-4161196-supplementary.pdf]

Biological and teratogenic evaluations of new nitrogen heterocycles for anticancer therapy

Jéssica Celerino dos Santos[1], Josival Emanuel Ferreira Alves[2], Rafael David Souto de Azevedo[1], Josefa Gerlane da Silva[1], Maria Regina de Oliveira Silva[1], Lucia Patrícia Bezerra Gomes da Silva[1], Caio Victor Silva Soares[3], Jamire Muriel da Silva[4]; Nabuêr Francieli da Silva[1], Jamerson Ferreira de Oliveira[3*], Maria do Carmo Alves de Lima[4], Ricardo Olímpio de Moura[5], Sinara Mônica Vitalino de Almeida [1,2,3]

[1] Molecular Biology Laboratory, University of Pernambuco (UPE), Multicampi Garanhuns, Garanhuns, PE, Brazil;

[2] Keizo Asami Immunopathology Laboratory (LIKA), Federal University of Pernambuco, Recife, PE, Brazil;

[3] University of International Integration of Afro-Brazilian Lusophony (UNILAB), Institute of Health Sciences, 62790-970, Redenção, CE, Brazil;

[4] Chemistry and Therapeutic Innovation Laboratory (LQIT), Department of Antibiotics, Federal University of Pernambuco, Recife, PE, Brazil;

[5] Department of Pharmacy, Laboratory of Synthesis and Vectorization of Molecules, State University of Paraíba (UEPB), Campus Campina Grande, 58429-500, PB, Brazil.

SUPPLEMENTARY MATERIAL

Figure S1. Proposal of the mechanism for all derivatives and spontaneous cyclization for 3a.

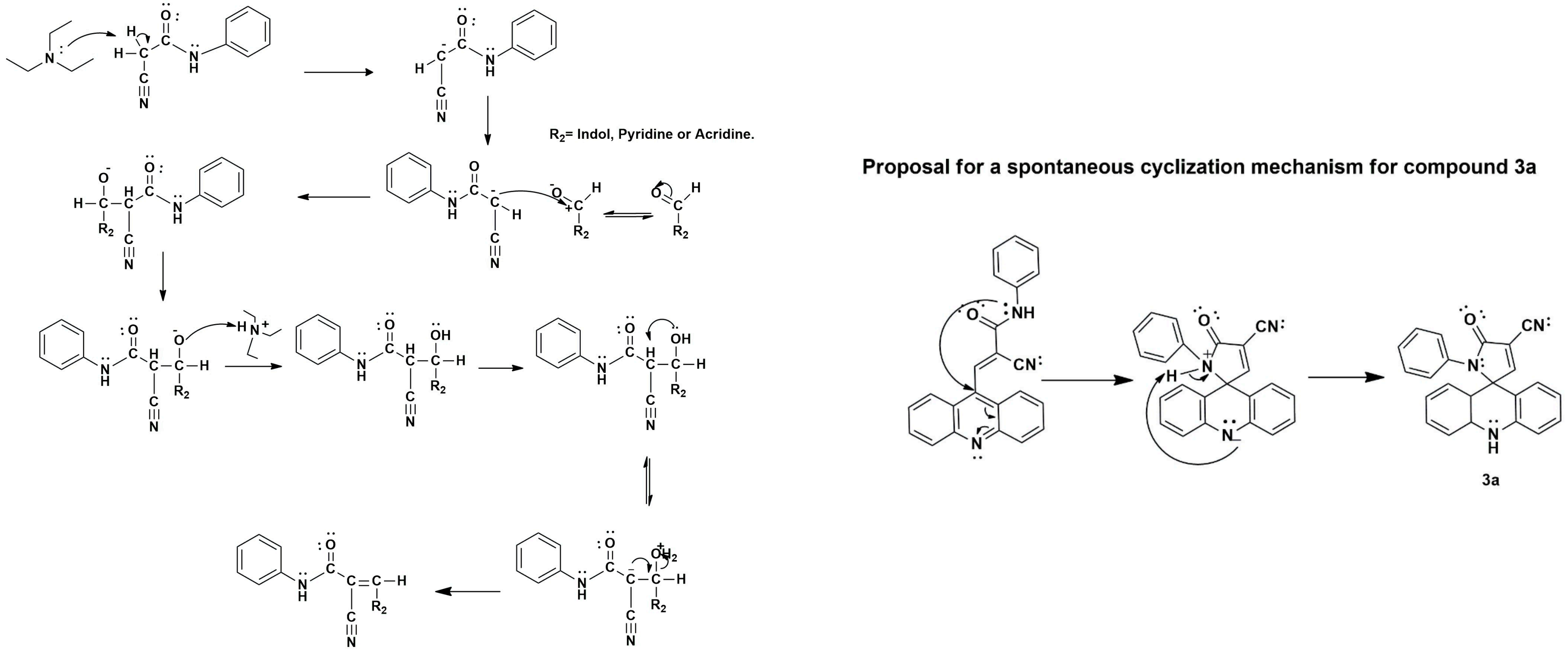

In this mechanism, triethylamine (TEA) is used to deprotonate the active methylene carbon located between the cyano and carbonyl groups. This carbon possesses an acidic character due to the electronic effects of the neighboring groups, allowing the proton to be easily abstracted by TEA to form a carbanion. Subsequently, this carbanion performs a nucleophilic attack on the carbonyl carbon of the aldehyde, resulting in a Knoevenagel-type condensation.

The protonated triethylamine then transfers its proton to the aldehyde oxygen, forming a hydroxyl group and regenerating the base. Dehydration follows, where the hydroxyl group and a geminal proton are eliminated as H_2O (leaving group), leading to the formation of a double bond. However, for one specific

derivative 3a undergoes spontaneous cyclization as following: Due to the strongly electrophilic nature of the carbon at position 9 of the acridine ring, it undergoes an intramolecular attack by the electron pair of the NH group of the amide, expelling the electrons to the N of the acridine ring, which is able to capture the proton from the NH group of the amide, leading to a spiro ring with lower energy than its open isomer.

Figure S2. ^{1}H-NMR spectrum (DMSO) of derivative **3a** (ACMD)

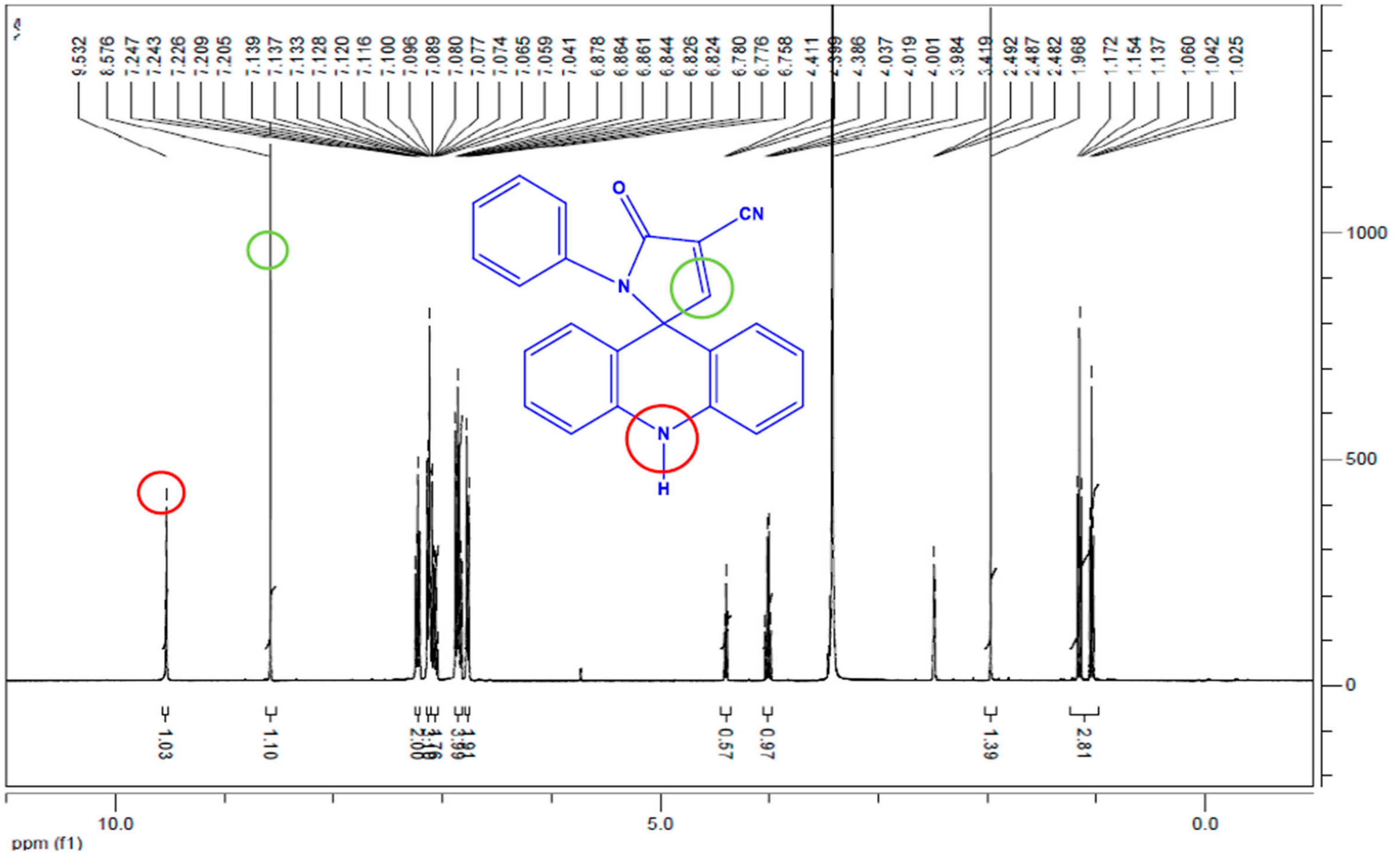

Figure S3. ^{13}C-NMR spectrum (DMSO) of derivative **3a (**ACMD)

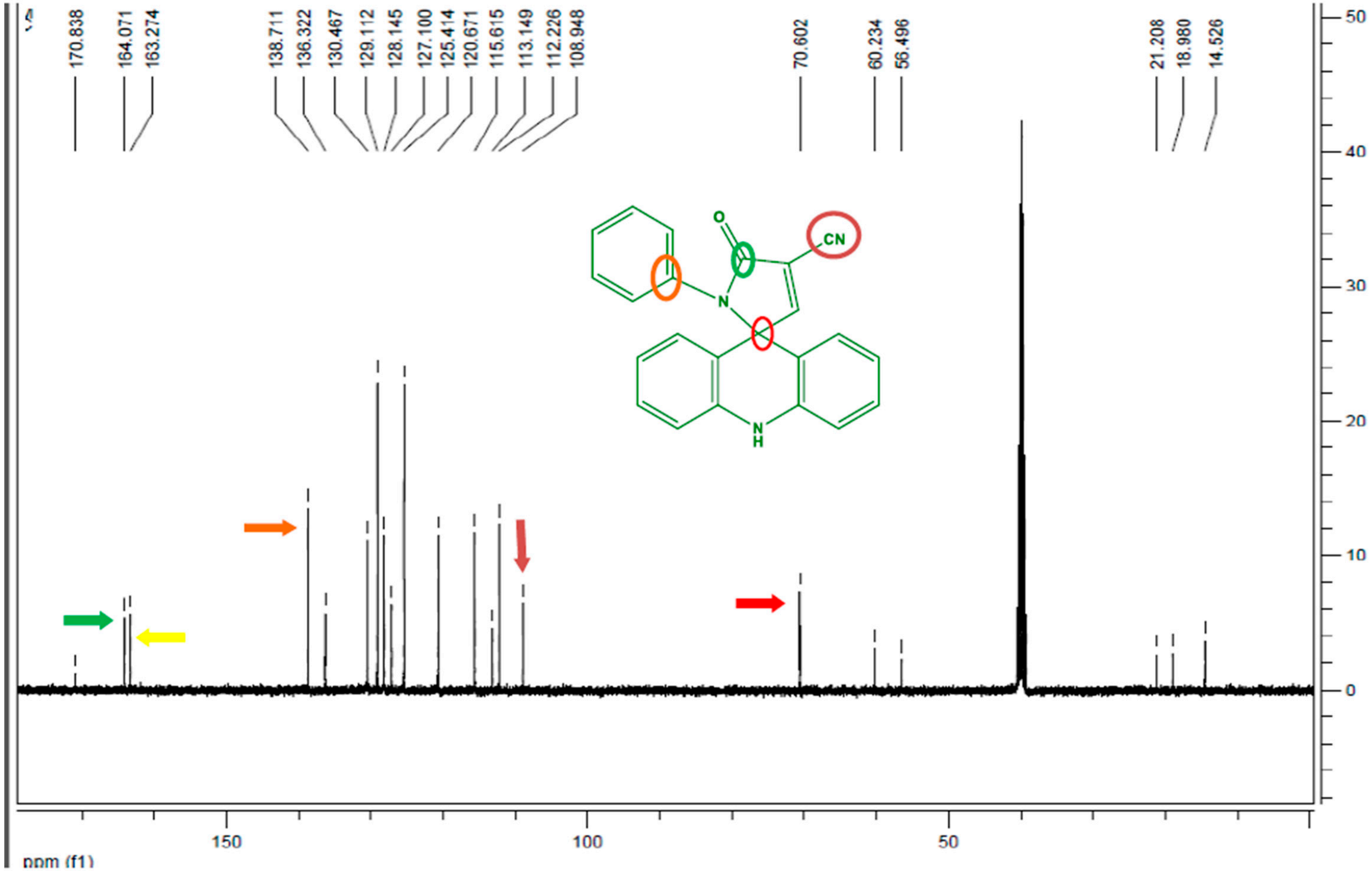

Figure S4. Infrared spectrum of derivative **3a (**ACMD)

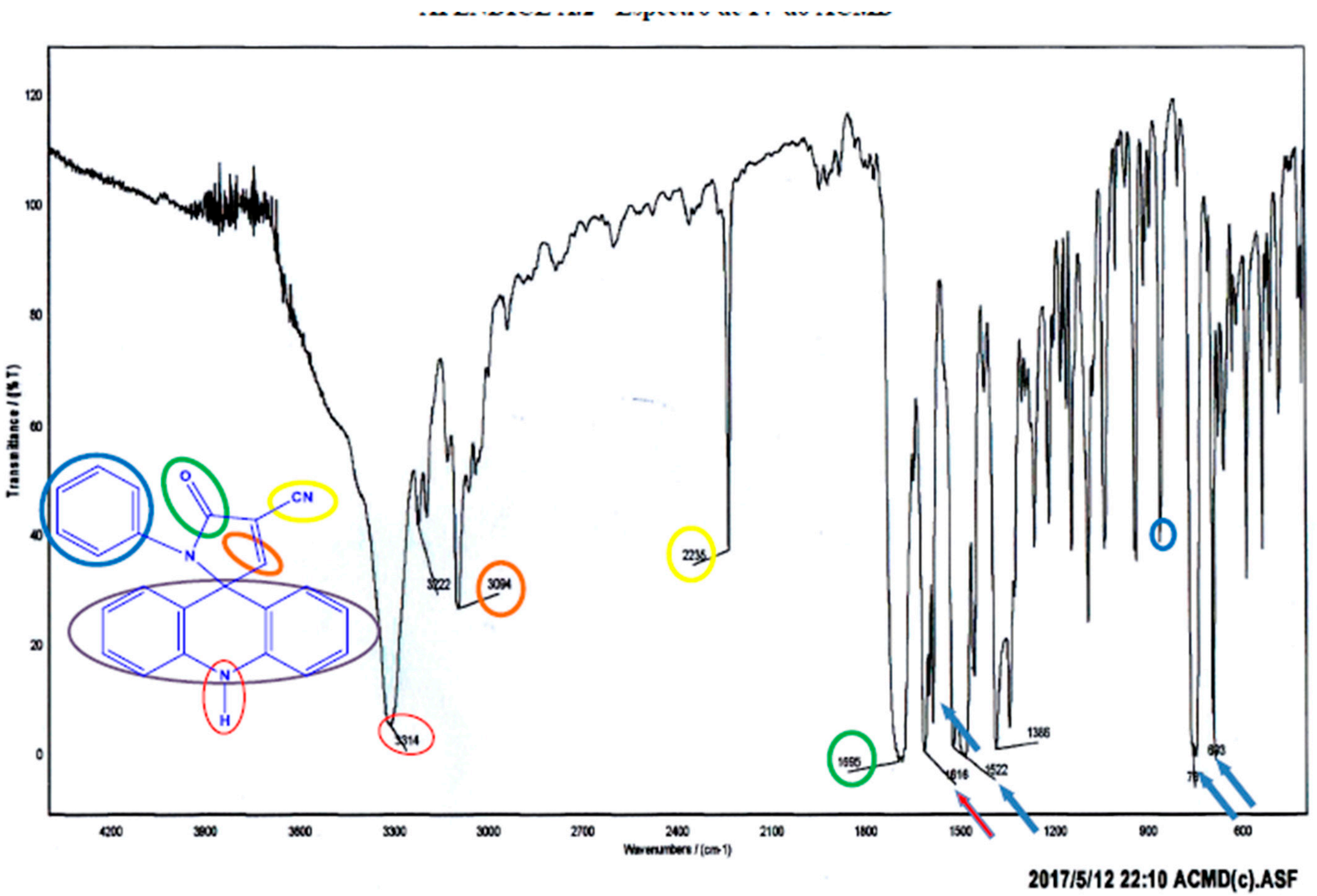

Figure S5. Mass spectrum of derivative **3a (**ACMD)

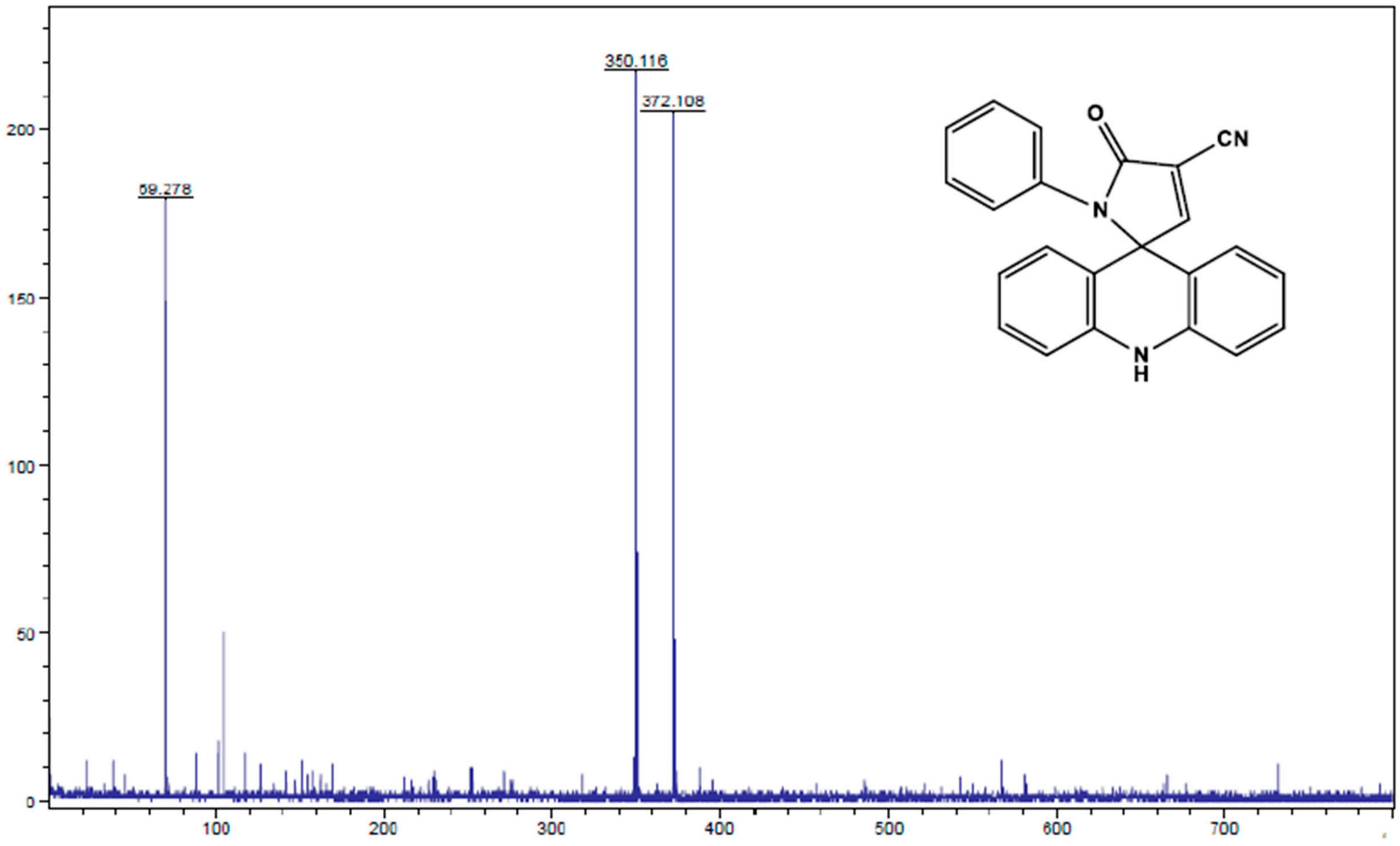

Figure S6. ^{1}H-NMR spectrum (DMSO) of derivative **3b (**QAMD)

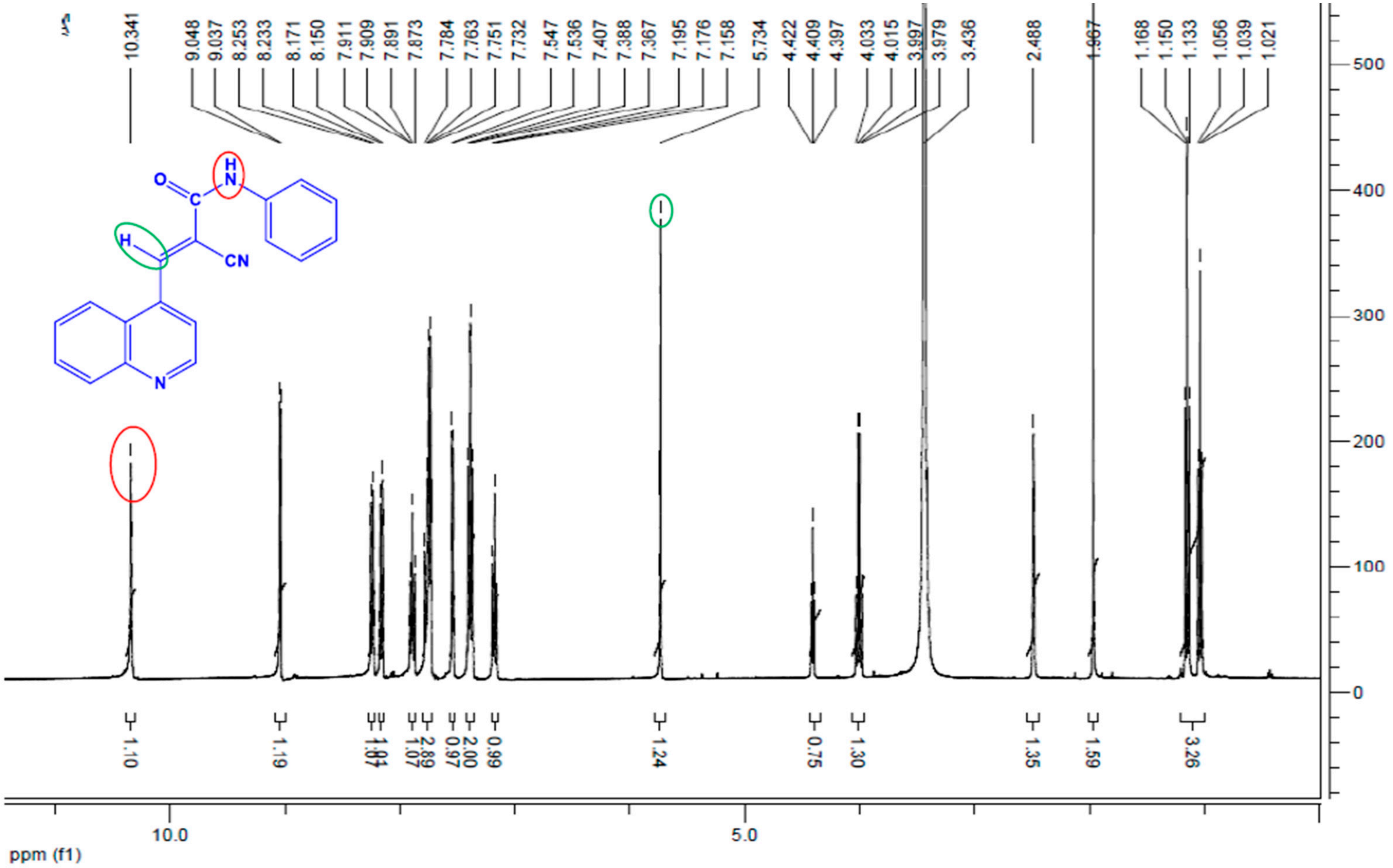

Figure S7. ^{13}C-NMR spectrum (DMSO) of derivative **3b (**QAMD)

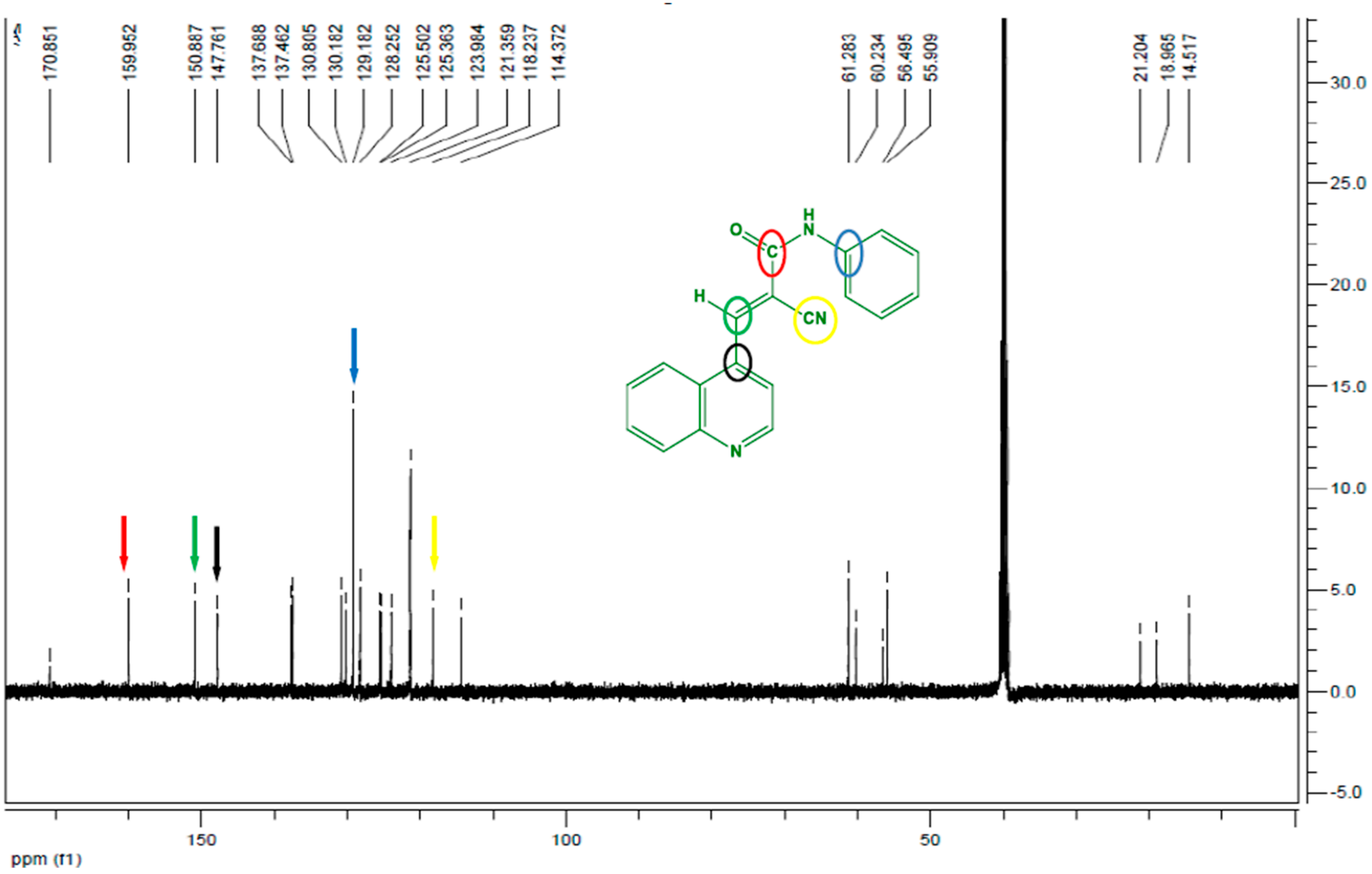

Figure S8. Infrared spectrum of derivative **3b** (QAMD)

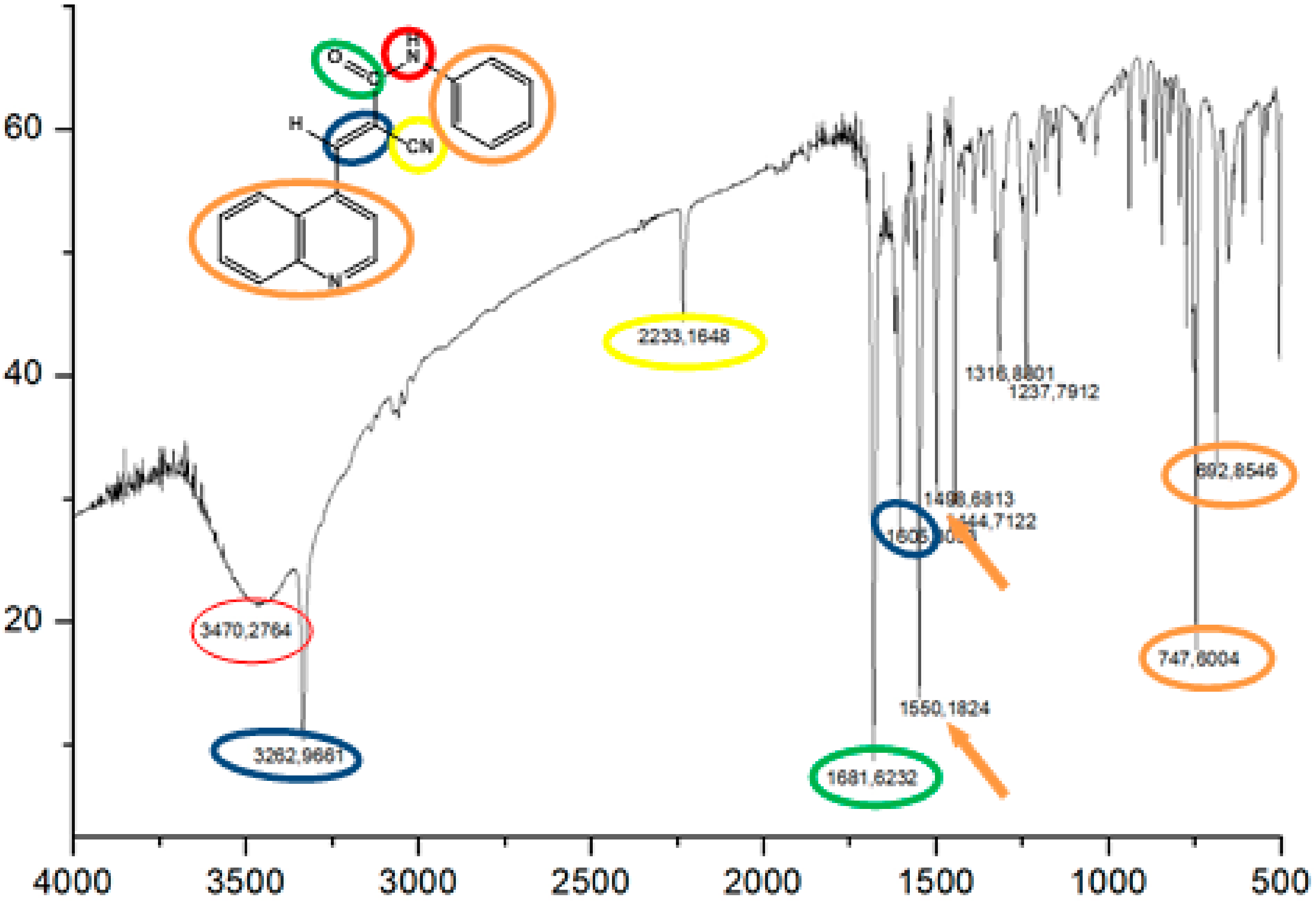

Figure S9. Mass spectrum of derivative **3b (**QAMD)

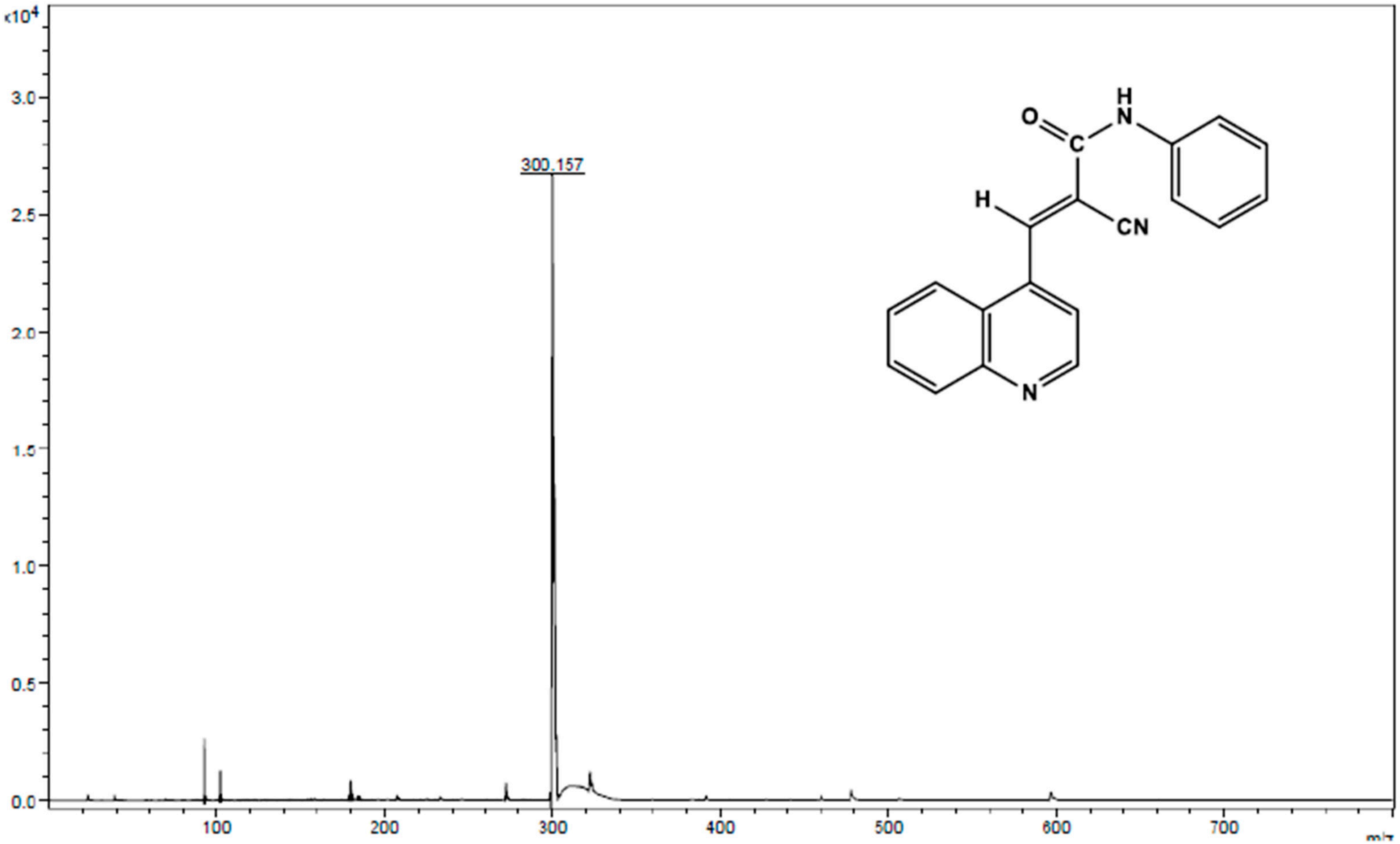

Figure S10. ^{1}H-NMR spectrum (DMSO) of derivative **3c (**ICMD)

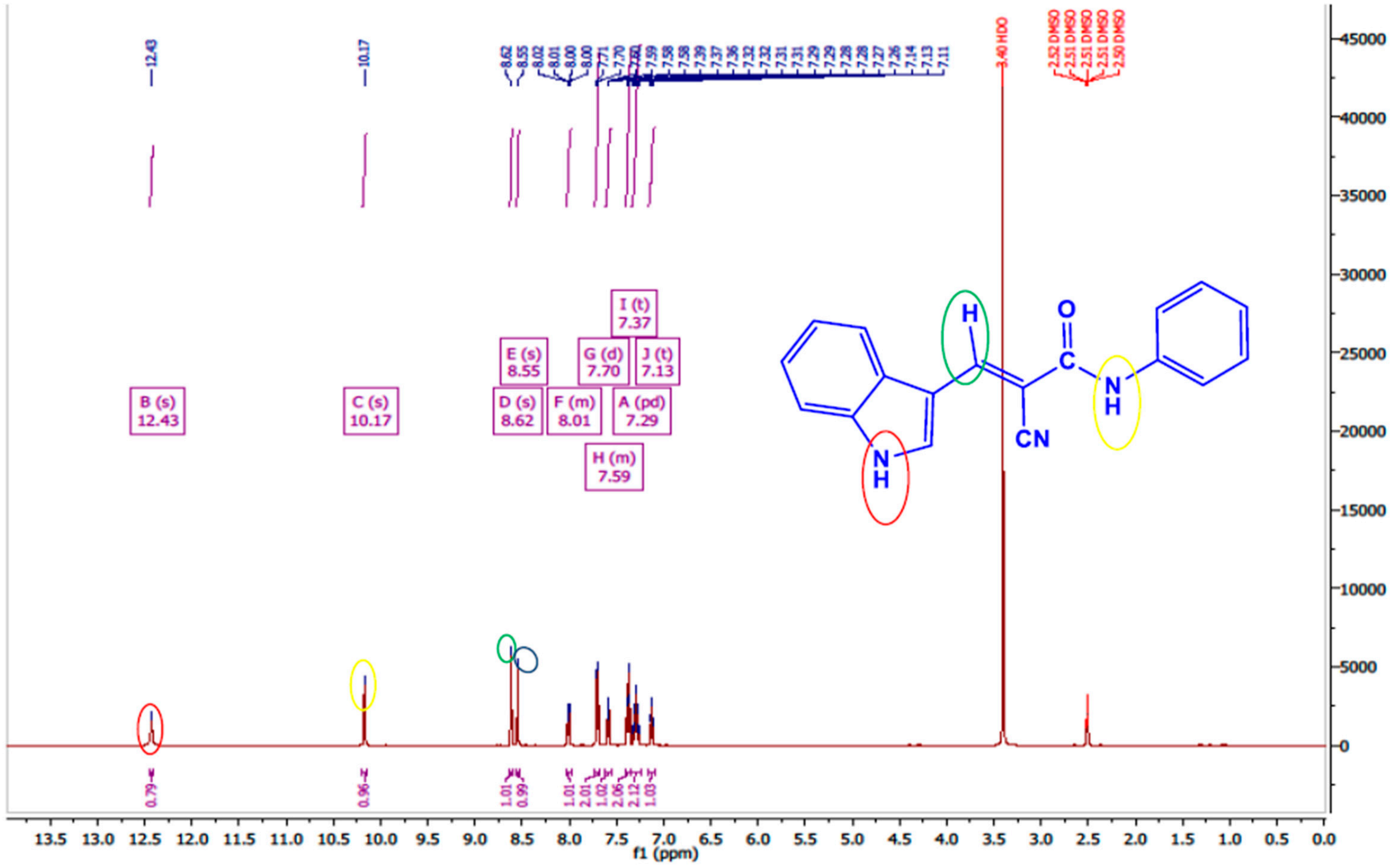

Figure S11. ^{13}C-NMR spectrum (DMSO) of derivative **3c (**ICMD)

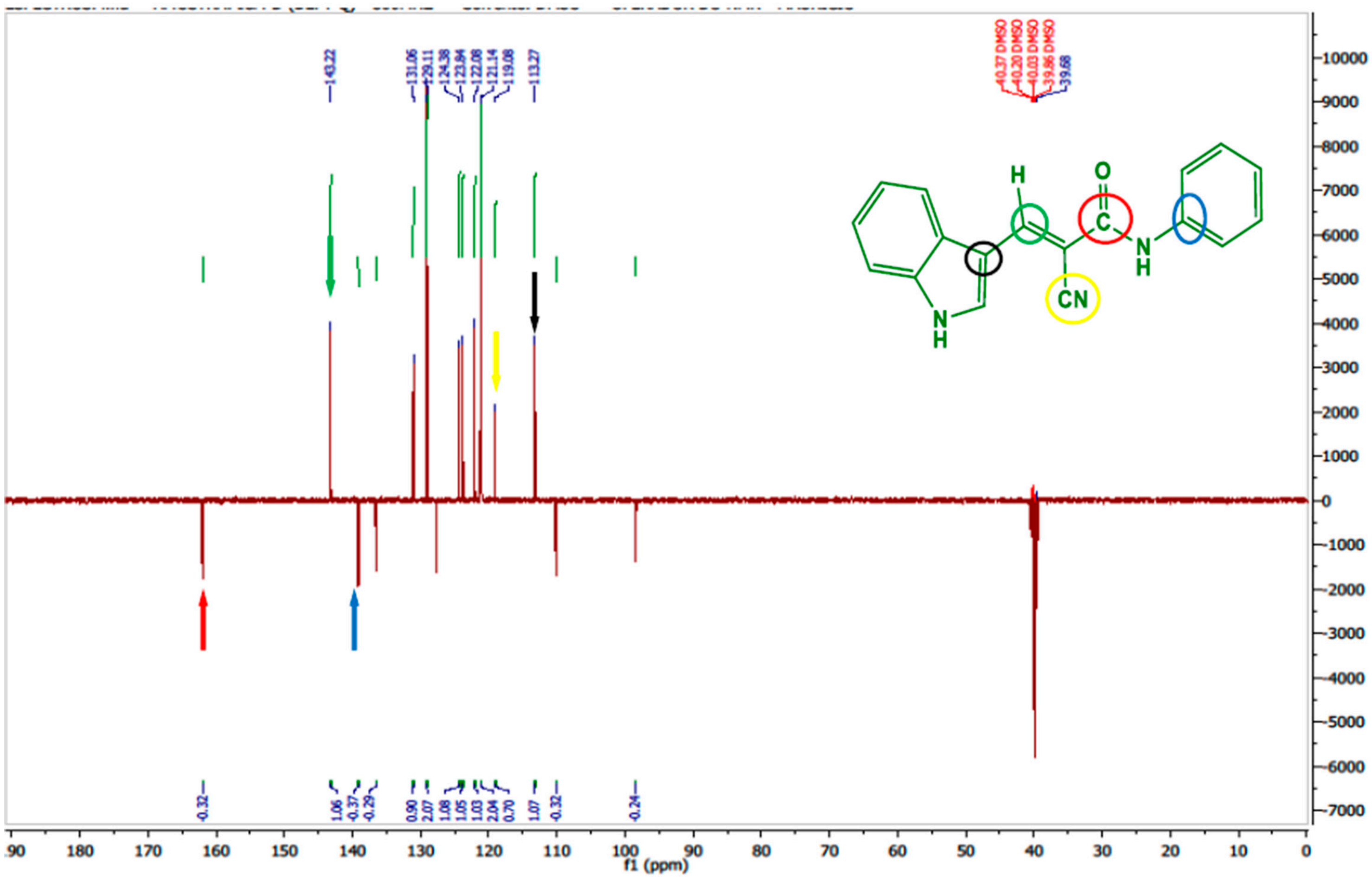

Figure S12. Infrared spectrum of derivative **3c** (ICMD)

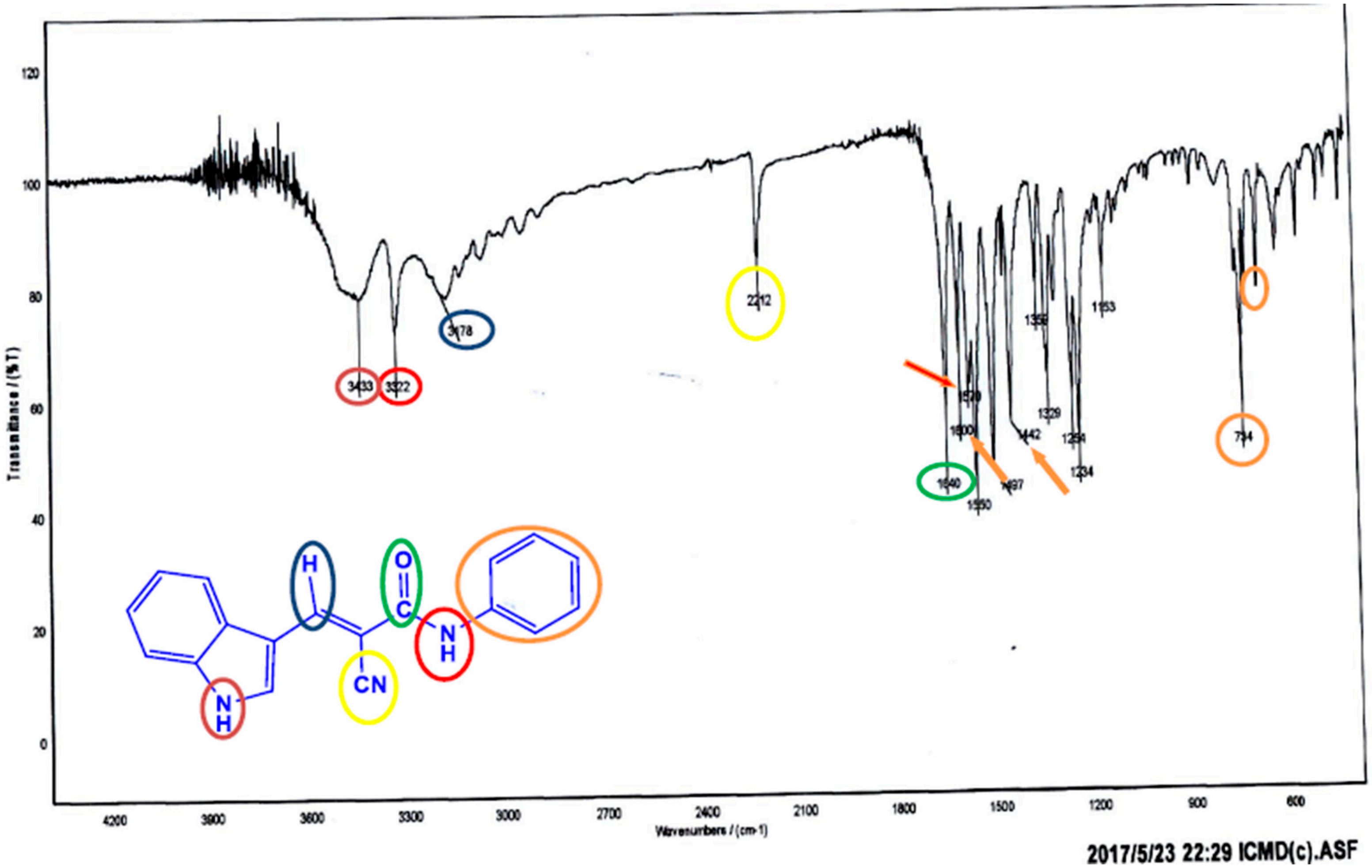

Figure S13. Mass spectrum of derivative **3c (**ICMD)

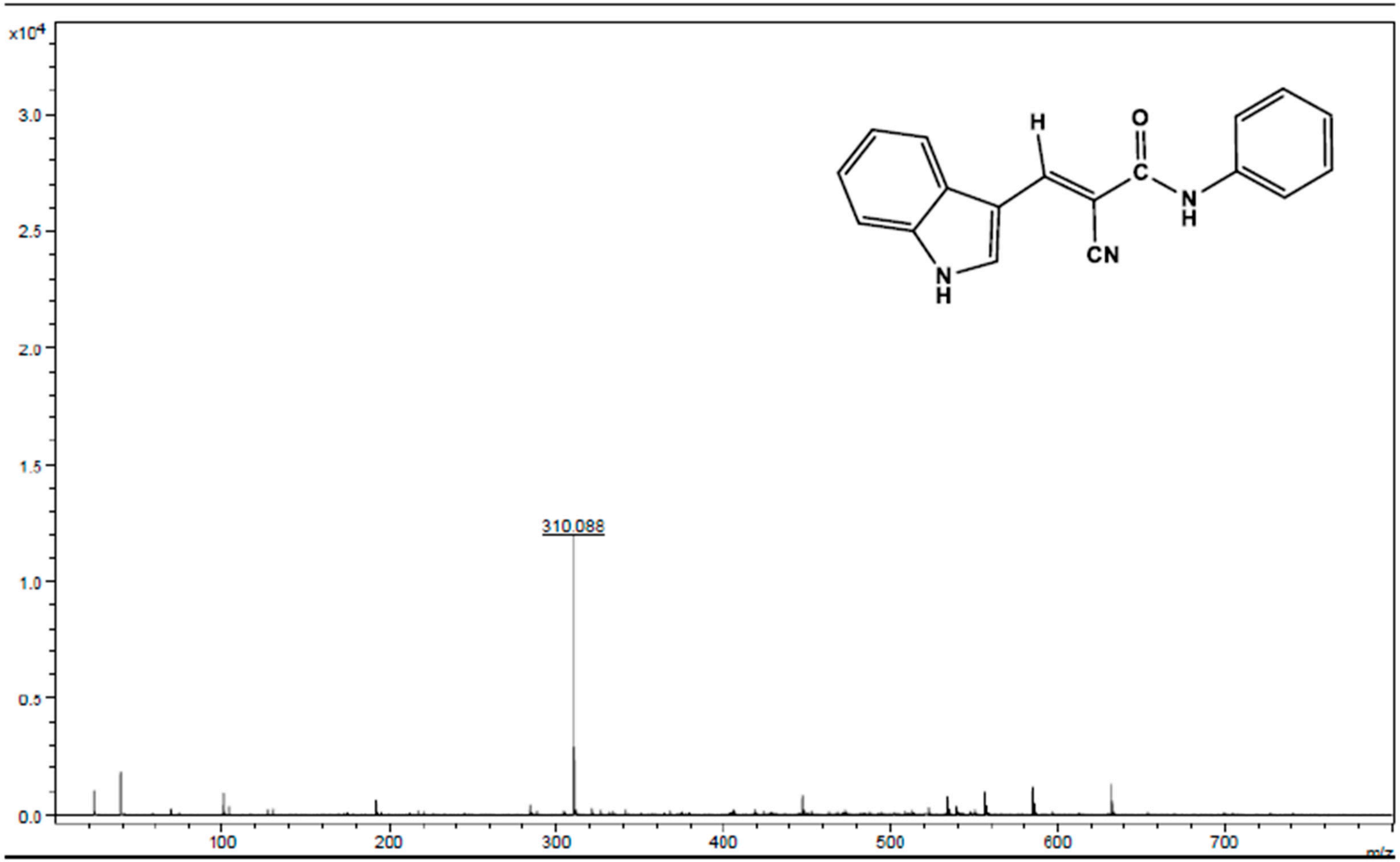

Figure S14. ^{1}H-NMR spectrum (DMSO) of derivative **3d (**PAMD)

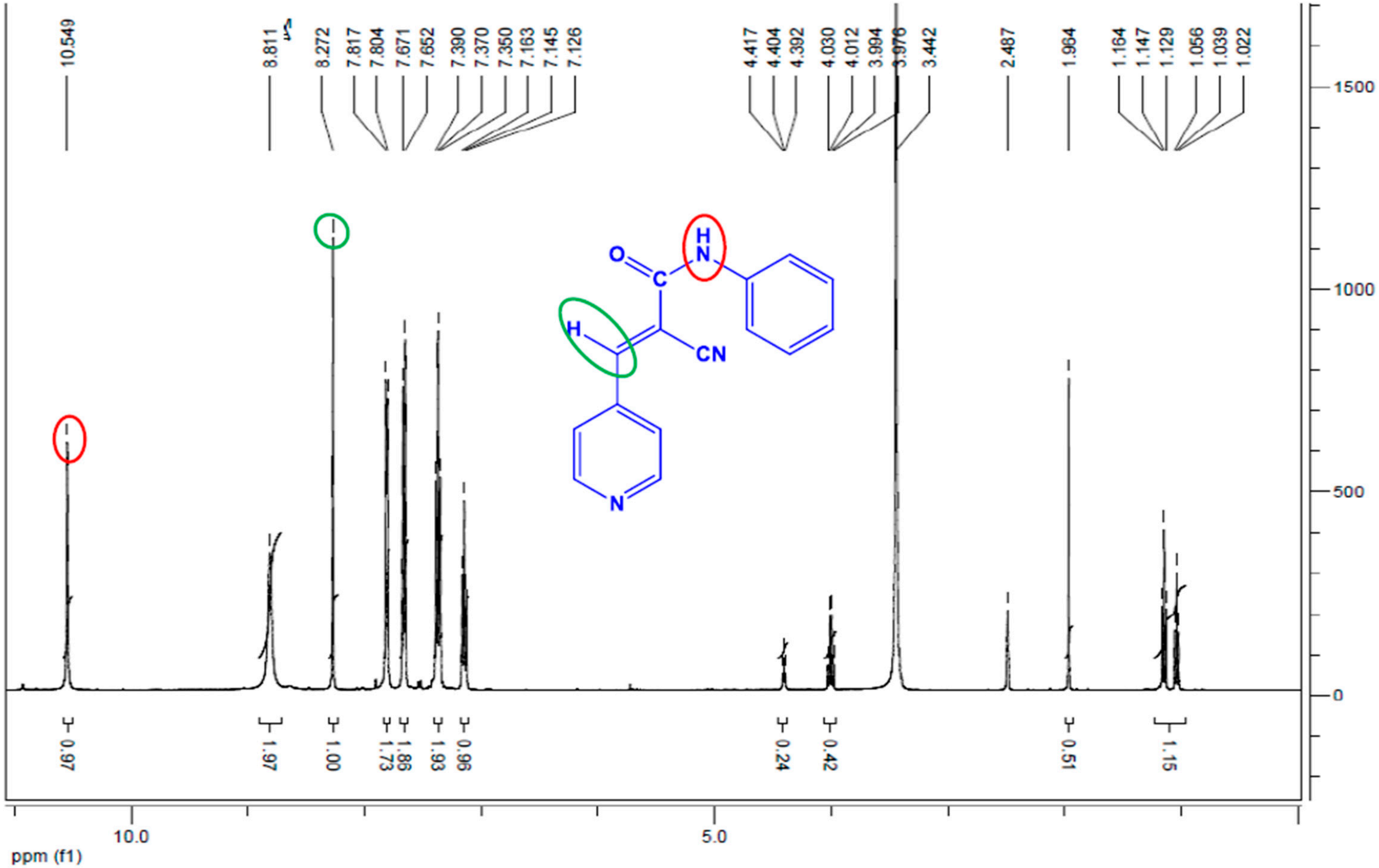

Figure S15. ^{13}C-NMR spectrum (DMSO) of derivative **3d (**PAMD)

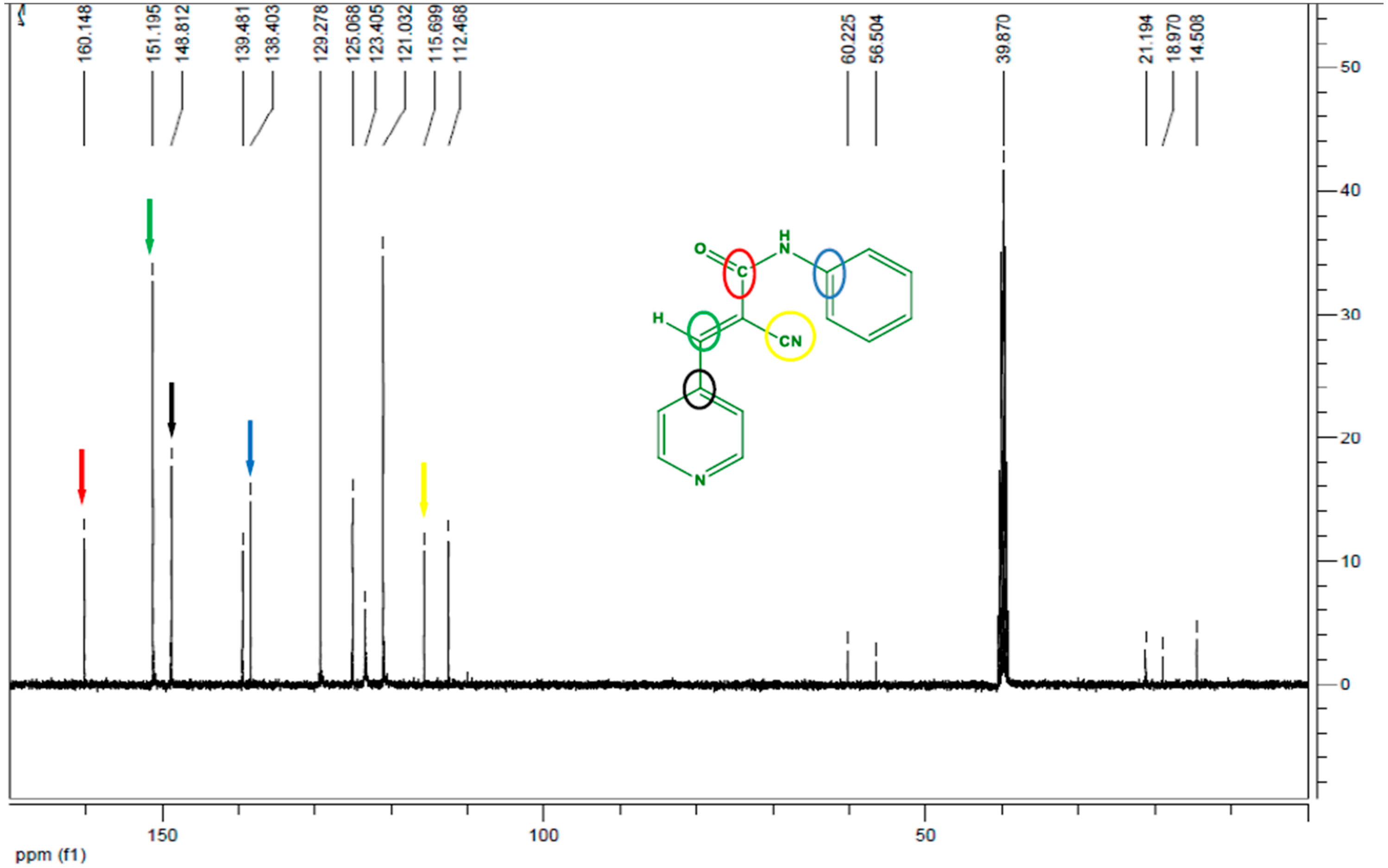

Figure S16. Infrared spectrum of derivative **3d** (PAMD)

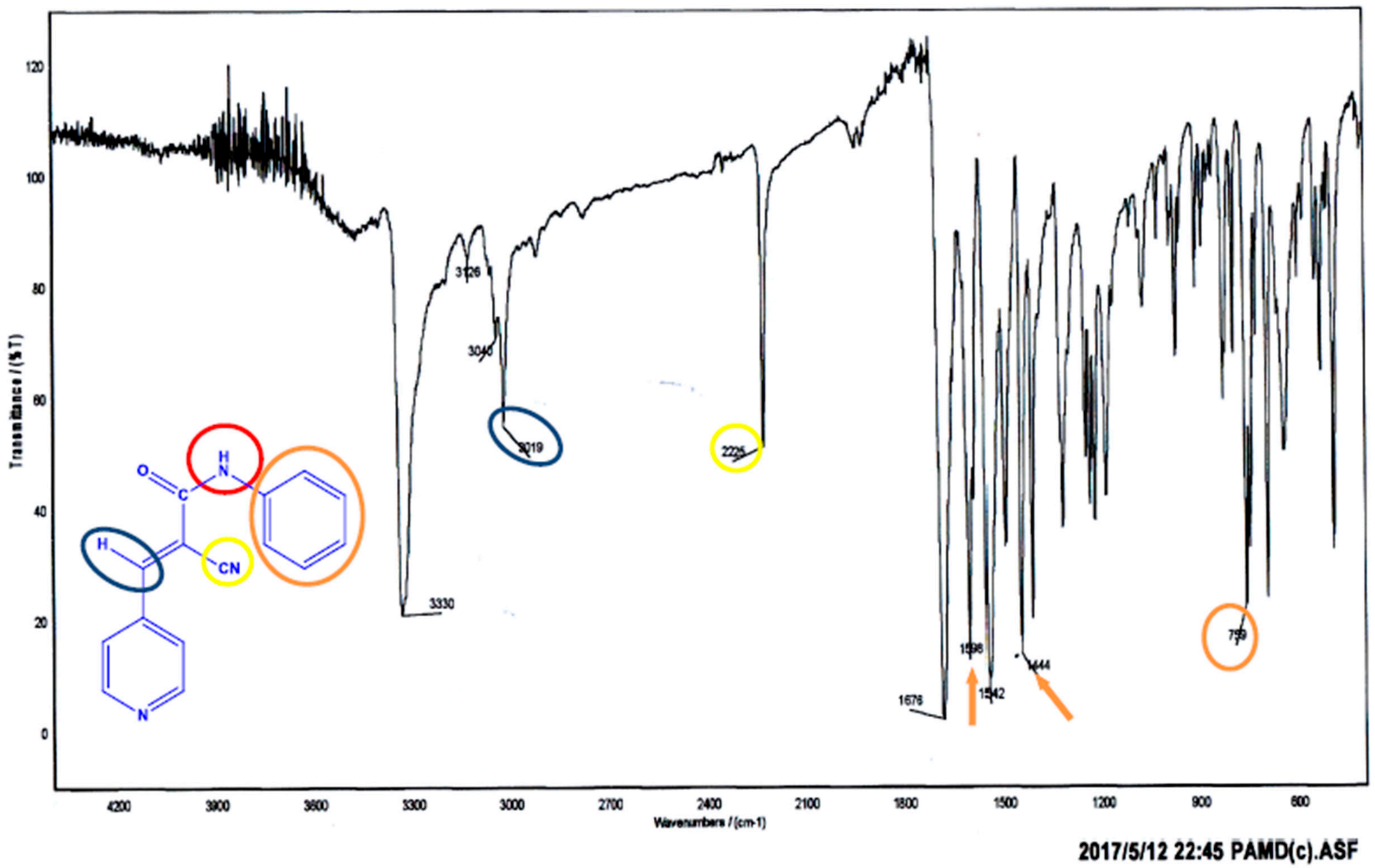

Figure S17. Mass spectrum of derivative **3d (**PAMD)

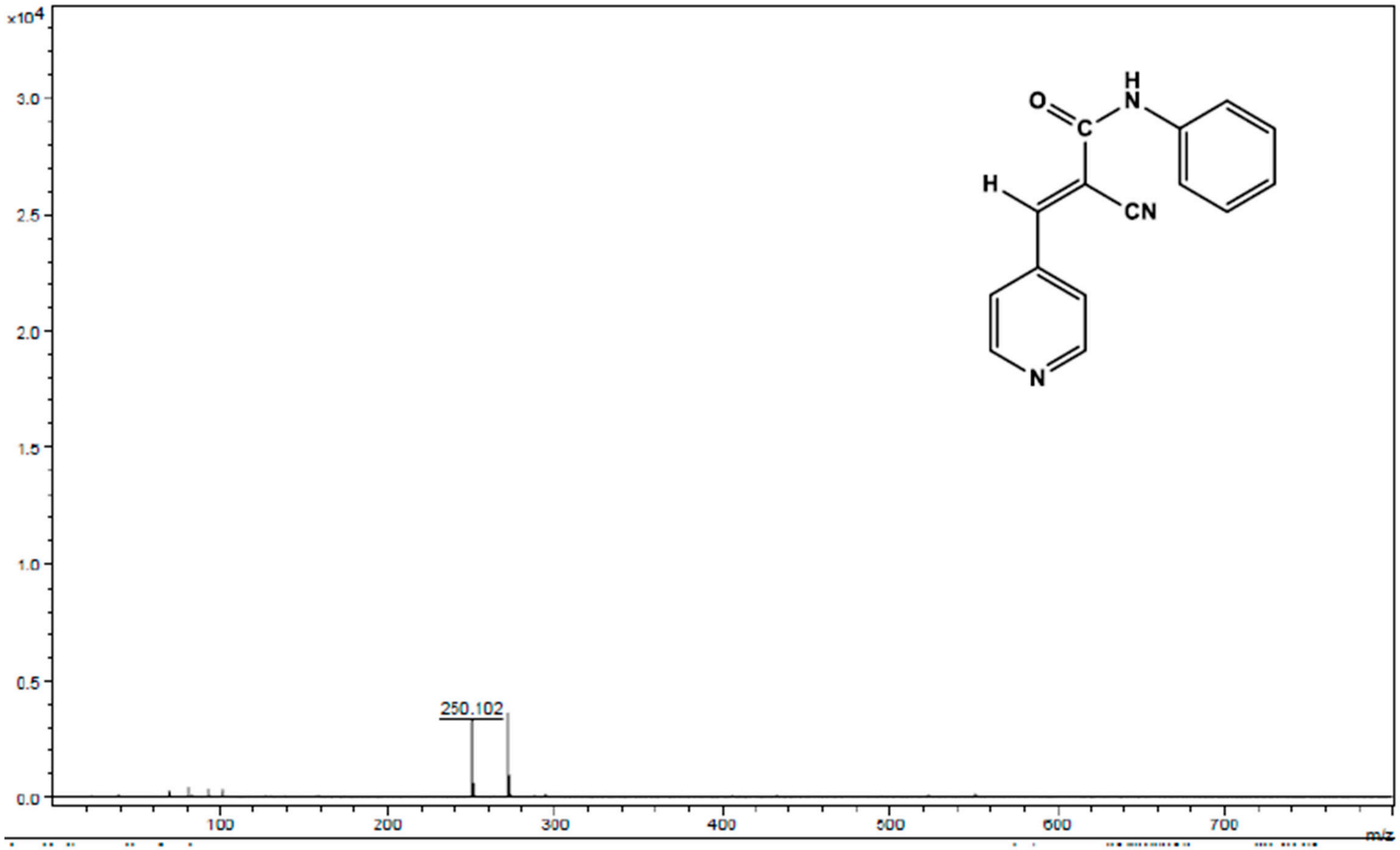

Figure S18. (A) Absorption spectra of **3a, 3b, 3c and 3d** in the presence of increasing amounts of ssDNA. [DNA] = 0, 10, 20, 40, 60, 80 and 100 µM. The arrows (↑), (↓), (→) and (←) refer to the hyperchromic, hypochromic, bathochromic and hypsochromic effects, respectively. (B) Insertion: corresponding to the [DNA] / (Ea - Ef) graph as a function of DNA concentration, as determined from spectral data.

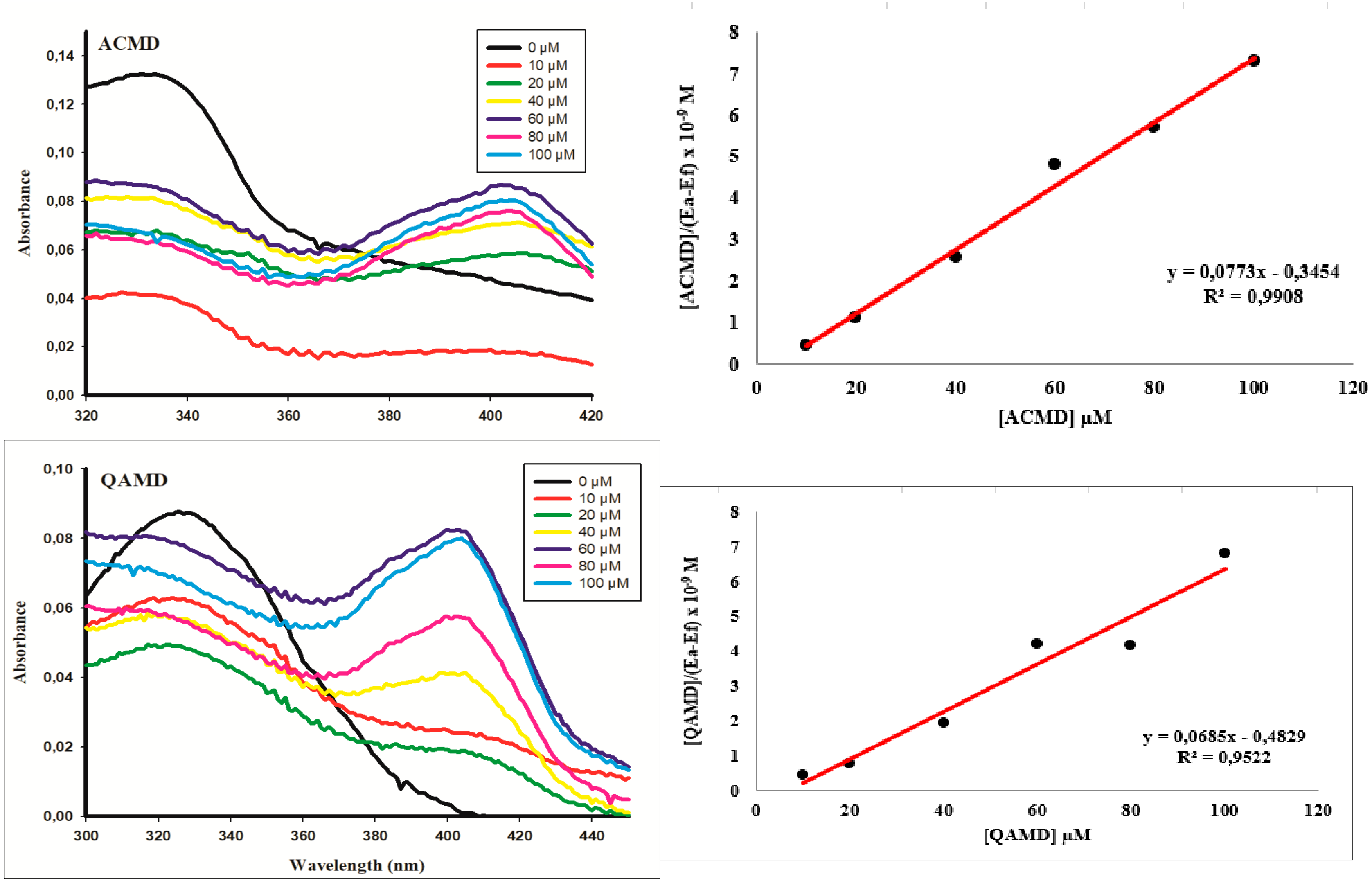

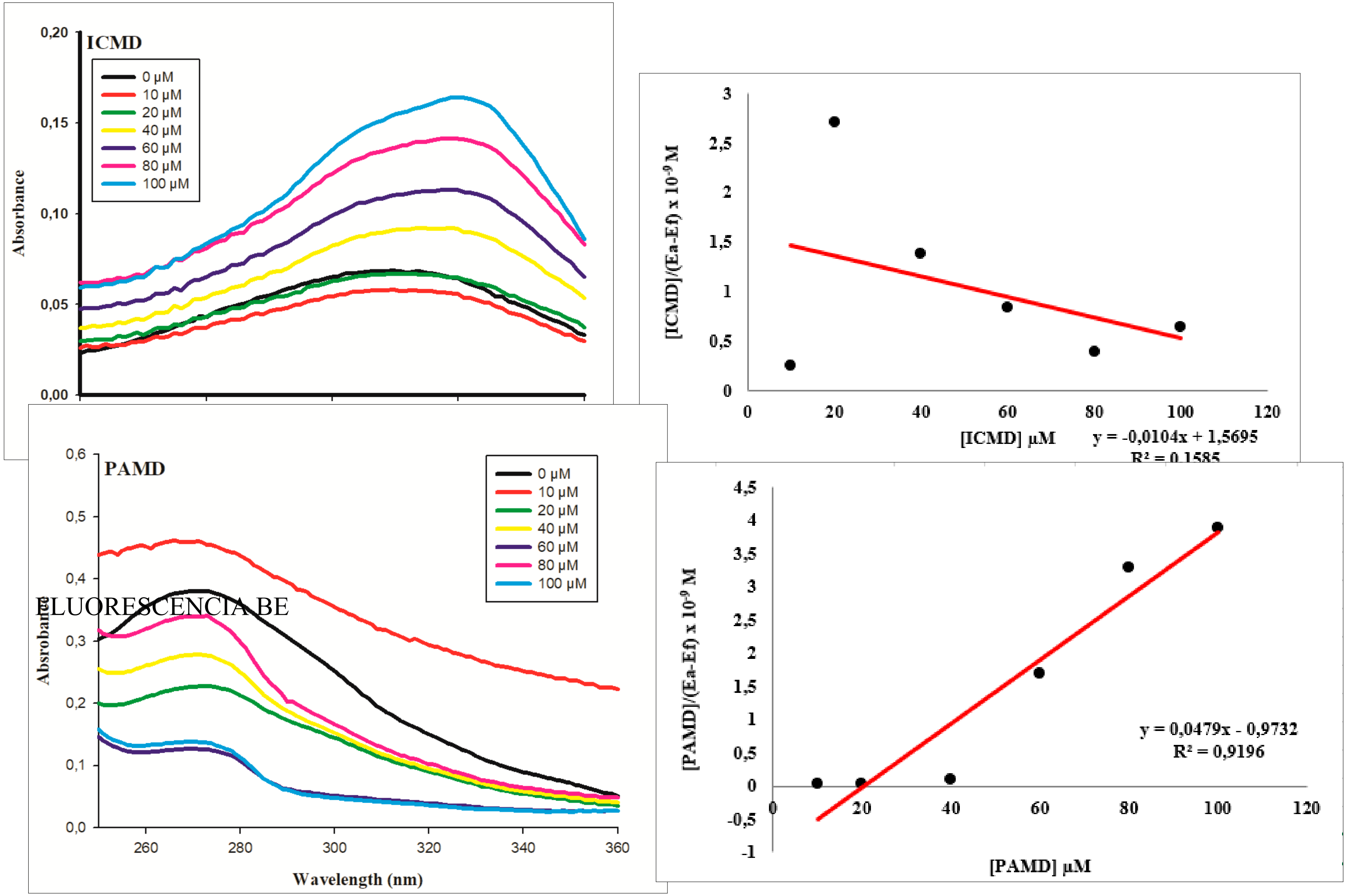

ICMD
Absorbance
0,20
0,15
0,10
0,05
0,00
0 µM
10 µM
20 µM
40 µM
60 µM
80 µM
100 µM
[ICMD]/(Ea-Ef) x 10^-9 M
[ICMD] µM
y = -0,0104x + 1,5695
R² = 0,1585
PAMD
Absrobance
FLUORESCENCIA BE
0,6
0,5
0,4
0,3
0,2
0,1
0,0
260
280
300
320
340
360
Wavelength (nm)
[PAMD]/(Ea-Ef) x 10^-9 M
[PAMD] µM
y = 0,0479x - 0,9732
R² = 0,9196

Figure S19. (A) Emission spectra of BE (10 µM) free (——) and bound to ssDNA (100 µM) in the absence (——) and in the presence of ACMD,QAMD, ICMD and PAMD. The arrow (↓) shows the decrease in intensity over the increase in the concentration of the complexes (10, 20, 40, 60, 80 and 100 µM) at room temperature. (B) Inserted: Stern-Volmer Suppression Graph of the relative suppression intensity (F0/F) as a function of the concentration of the derivative, as determined from the spectral data.

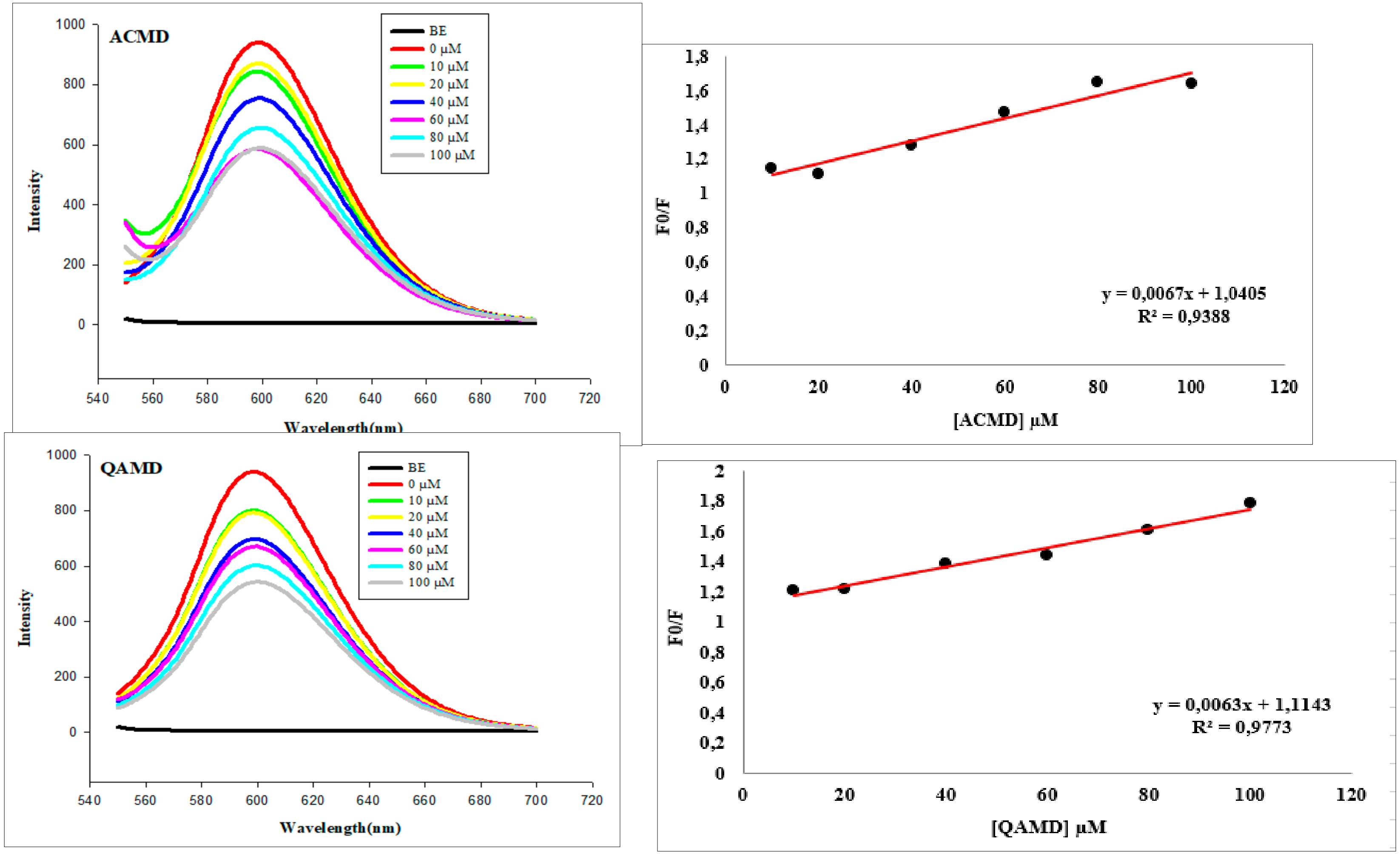

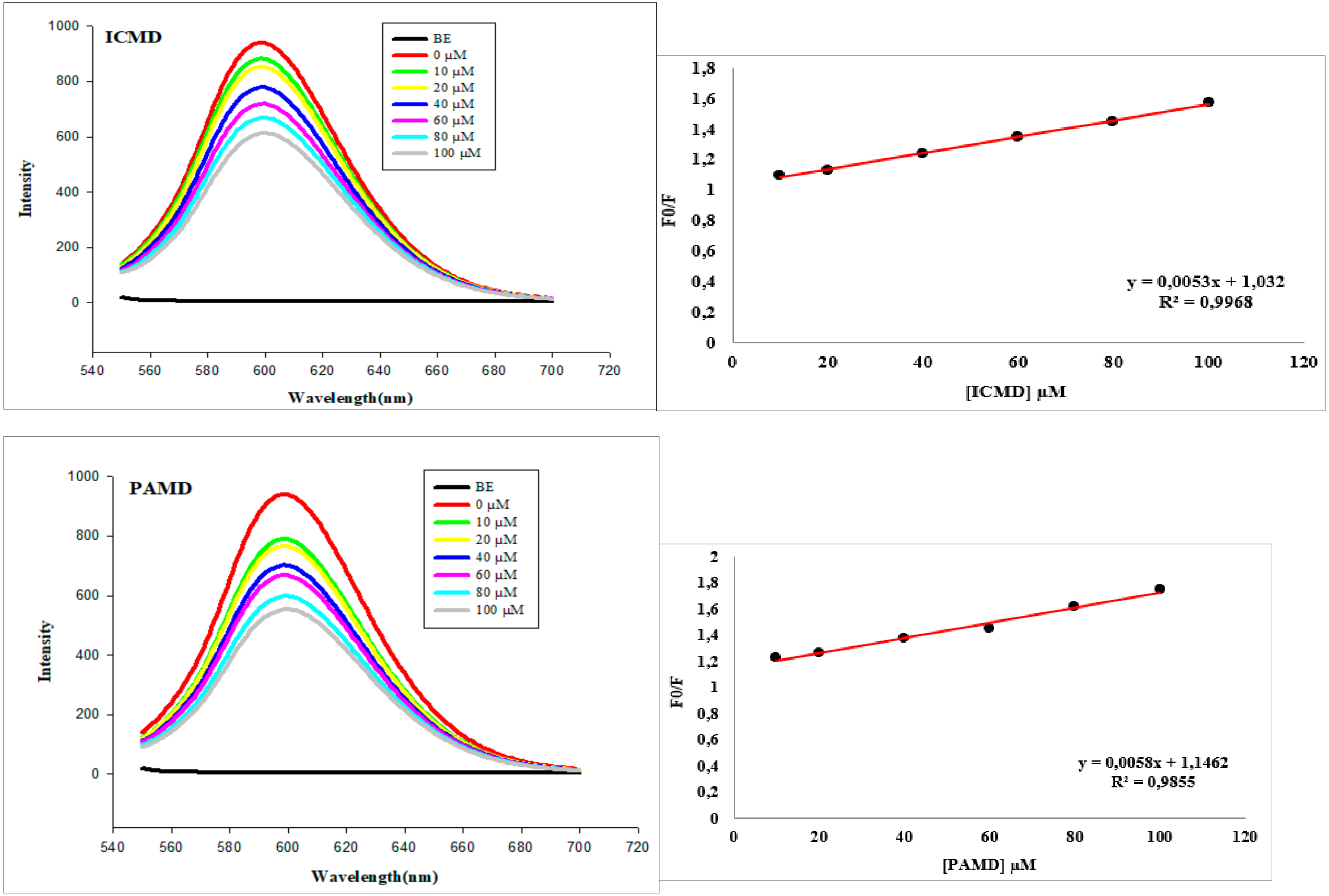
ICMD
Intensity
Wavelength(nm)
BE
0 µM
10 µM
20 µM
40 µM
60 µM
80 µM
100 µM
F0/F
[ICMD] µM
y = 0,0053x + 1,032
R² = 0,9968
PAMD
Intensity
Wavelength(nm)
BE
0 µM
10 µM
20 µM
40 µM
60 µM
80 µM
100 µM
F0/F
[PAMD] µM
y = 0,0058x + 1,1462
R² = 0,9855

Figure S20: Structure of **3a** (ACMD), **3b** (QAMD), **3c** (ICMD), and **3d** (PAMD) derivatives along with their respective modes of interaction with DNA and amino acid residues at the targets.

Derivative	Alvo: 1G3X	Alvo: 5GWK
**3a**	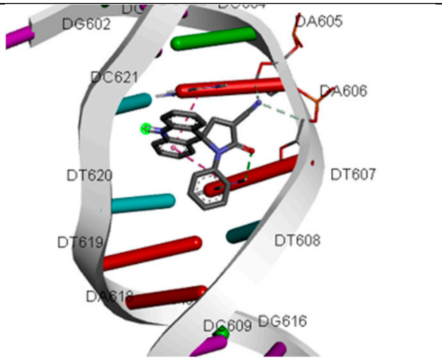	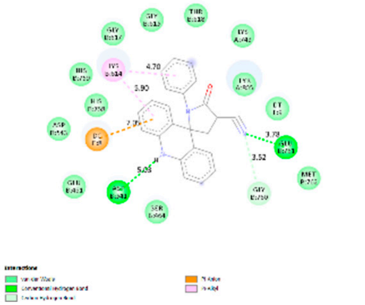
**3b**	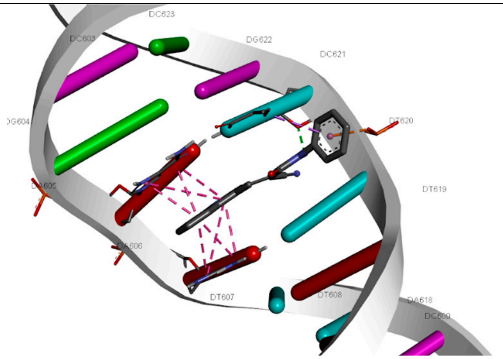	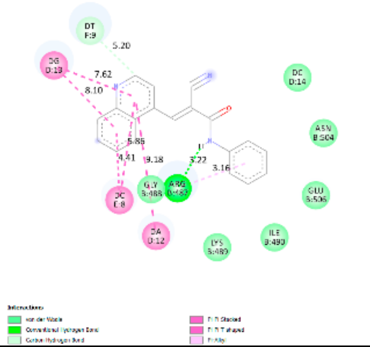

**3c**	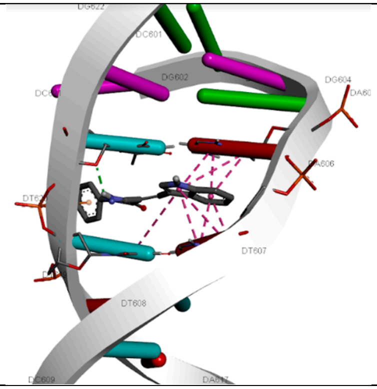	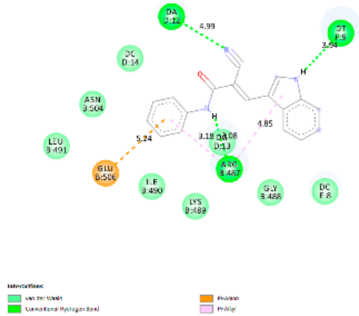
**3d**	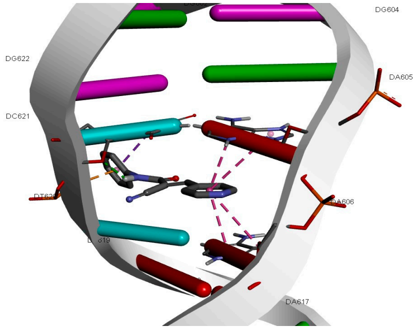	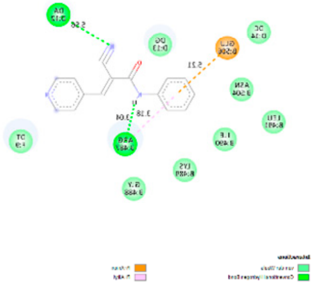
